# From knowledge silos to integrated insights: building a cardiovascular medication knowledge graph for enhanced medication knowledge retrieval, relationship discovery, and reasoning

**DOI:** 10.3389/fcvm.2025.1526247

**Published:** 2025-04-28

**Authors:** Hongzhen Cui, Xiaoyue Zhu, Wei Zhang, Meihua Piao, Yunfeng Peng

**Affiliations:** ^1^ School of Computer and Communication Engineering, University of Science and Technology Beijing, Beijing, China; ^2^ Key Laboratory of Computing Power Network and Information Security, Ministry of Education, Shandong Computer Science Center (National Supercomputer Center in Jinan), Qilu University of Technology (Shandong Academy of Sciences), Jinan, China; ^3^ Shandong Provincial Key Laboratory of Computing Power Internet and Service Computing, Shandong Fundamental Research Center for Computer Science, Jinan, China; ^4^ School of Nursing, Chinese Academy of Medical Sciences, Peking Union Medical College, Beijing, China

**Keywords:** cardiovascular medication knowledge, expert knowledge graph, multidimensional relationships, ontology construction, knowledge discover and reasoning, EKG-CMG

## Abstract

**Background:**

Cardiovascular diseases are diverse, intersecting, and characterized by multistage complexity. The growing demand for personalized diagnosis and treatment poses significant challenges to clinical diagnosis and pharmacotherapy, increasing potential medication risks for doctors and patients. The Cardiovascular Medication Guide (CMG) demonstrates distinct advantages in managing cardiovascular disease, serving as a critical reference for front-line doctors in prescription selection and treatment planning. However, most medical knowledge remains fragmented within written records, such as medical files, without a cohesive organizational structure, leading to an absence of clinical support from visualized expert knowledge systems.

**Purpose:**

This study aims to construct a comprehensive Expert Knowledge Graph of Cardiovascular Medication Guidelines (EKG-CMG) by integrating unstructured and semi-structured Cardiovascular Medication Knowledge (CMK), including clinical guidelines and expert consensus, to create a visually integrated cardiovascular expert knowledge system.

**Methods:**

This study utilized consensus and guidelines from cardiovascular experts to organize and manage structured knowledge. BERT and knowledge extraction techniques capture drug attribute relationships, leading to the construction of the EKG-CMG with fine-grained information. The Neo4j graph database stores expert knowledge, visualizes knowledge structures and semantic relationships, and supports retrieval, discovery, and reasoning of knowledge about medication. A hierarchical-weighted, multidimensional relational model to mine medication relationships through reverse reasoning. Experts participated in an iterative review process, allowing targeted refinement of expert medication knowledge reasoning.

**Results:**

We construct an ontology encompassing 12 cardiovascular “medication types” and their “attributes of medication types”. Approximately 15,000 entity-relationships include 22,475 medication entities, 2,027 entity categories, and 3,304 relationships. Taking beta-blockers (β) as an example demonstrates the complete process of ontology to knowledge graph construction and application, encompassing 41 AMTs, 1,197 entity nodes, and 1,351 relationships. The EKG-CMG can complete knowledge retrieval and discovery linked to “one drug for multiple uses,” “combination therapy,” and “precision medication.” Additionally, we analyzed the knowledge reasoning case of cross-symptoms and complex medication for complications.

**Conclusion:**

The EKG-CMG systematically organizes CMK, effectively addressing the “knowledge island” issues between diseases and drugs. Knowledge potential relationships have been exposed by leveraging EKG-CMG visualization technology, which can facilitate medication semantic retrieval and the exploration and reasoning of complex knowledge relationships.

## 1 Introduction

Research on cardiovascular treatment has continually advanced with time. Specifically, the application of beta-blockers has highlighted the essential effect of sympathetic nerve activity in regulating cardiac contraction (1). Today, bidirectional regulation of sympathetic nerve activity is possible. Concurrently, significant advancements have been made in understanding cardiac ion currents and acute ischemic diseases (2). The application of calcium channel blockers (CCB) has underscored the crucial role of calcium ions at the cellular level, particularly their involvement in necrosis and arrhythmias. The discovery, development, mechanisms of action, and treatment processes of these representative drugs have become integral to the history of cardiology. Undoubtedly, significant advancements in cardiovascular treatment include the use of angiotensin-converting enzyme inhibitors (3), statins (4), thrombolytic drugs (5), and novel antiplatelet agents (6). Notably, the use of beta-blockers has evolved from treating angina pectoris and hypertension to providing protective treatment post-myocardial infarction, followed by their contraindication in heart failure, and ultimately to their role as a crucial agent for primary prevention, exemplifying the transformative impact of drug innovation on cardiovascular care.

Most cardiovascular diseases are chronic diseases, and long-term medication treatment makes patients develop medication compliance. Targeted drug therapy has shown significant efficacy in symptom relief, disease progression delay, heart function improvement, and quality of life enhancement. Beta-blockers have an outstanding effect on age-related changes in elderly heart failure patients (7). Therefore, systematically organizing CMK, constructing a medication knowledge graph, and exploring medication information are crucial for guiding doctors and patients in disease prevention, treatment, symptom management, and prognosis care.

In the era of big data, knowledge graph construction technology is increasingly essential for large-scale knowledge management, mining, and intelligent reasoning applications. This technology utilizes graph models to represent unstructured and semi-structured knowledge, implementing knowledge semantics, data association, and data source expansion through the modeling of various instances, entities, their attributes, and relationships (8). By organizing and integrating dispersed knowledge based on relationships, this technology provides an intelligent solution to the “knowledge island” problem, enhancing knowledge and information management.

Recently, knowledge graph construction technology has been widely applied in medicine and biology (9, 10), offering crucial technical support for knowledge mining, integration, management, and sharing. However, these studies primarily focus on electronic medical records and general medical applications, including the modeling, optimization, and embedding of algorithms (11, 12) like named entity recognition (13), relationship classification (14), and entity disambiguation (15). Research and applications related to constructing the EKG-CMG, mining, and reasoning medication knowledge, and building precise expert knowledge graphs remain underdeveloped and face significant challenges.

### 1.1 Credibility of knowledge sources

“Drugs for the Heart, 7th Edition” was edited by Drs. Lionel H. Opie and Bernard J. Gersh. This book presents highly portable, up-to-date information on every drug class used to treat cardiovascular disease. It has been highly favored by cardiovascular disease doctors in Europe and America. This study utilizes the professional medication knowledge from the Chinese *Drugs for the Heart, 7th Edition* to conduct knowledge organization, modeling, algorithm design, and scientific storage, ensuring the acquisition and management of highly reliable knowledge sources. Additionally, the study incorporates reliable consensus data sources, including the *Chinese Guidelines for the Diagnosis and Treatment of Heart Failure 2024* (16) and the *Chinese Guidelines for the Prevention and Treatment of Hypertension 2024* (17), etc., to expand and validate the coverage and accuracy of the knowledge. As authoritative works in the cardiovascular field in China, references (16, 17) have attracted the attention of many readers at home and abroad.

### 1.2 Professionalism of knowledge sources

Using authoritative guidelines and expert consensus as data sources for constructing cardiovascular medication knowledge graphs ensures scientific rigor, professionalism, and accuracy. For instance, Zhao et al. (18) constructed a traditional Chinese medicine knowledge graph for diabetic nephropathy. However, there is a lack of comparable research in cardiovascular disease medication. In cardiovascular medication, professional guideline data offers highly reliable support for constructing the Expert Knowledge Graph of Cardiovascular Medication Guidelines (EKG-CMG). Although guidelines and consensus knowledge are continually updated and expanded, EKG-CMG includes an extensible interface and update functionality, enabling real-time integration of new medication knowledge.

### 1.3 Fine-grained management of entities and relationships in knowledge structures

Knowledge graph construction relies on ontology, which defines conceptual elements like classes, relationships, and attributes, presenting the knowledge system’s internal logic. Most existing medical knowledge graphs reuse pre-existing ontologies, extracting entities and relationships from unstructured texts to enrich and refine ontologies at the data level. However, this approach often leads to coarse granularity in concepts and relationships, limiting its effectiveness in clinical applications.

The CMK requires precise extraction of medication knowledge from unstructured and semi-structured texts, particularly in accurately identifying medication entities, attributes, and relationships to construct fine-grained ontologies for specific medication scenarios. This paper introduces an innovative ontology construction for specific CMK, maximizing the retention of native relationships in CMG, thus expanding the scope of existing medical knowledge graphs while preserving relationship diversity and data authenticity. Consequently, the EKG-CMG incorporates more relationship types while maintaining the crucial characteristics of the original data. This paper uses β-blockers as a case study to illustrate and detail the ontology construction process and description information.

**Figure 1:**
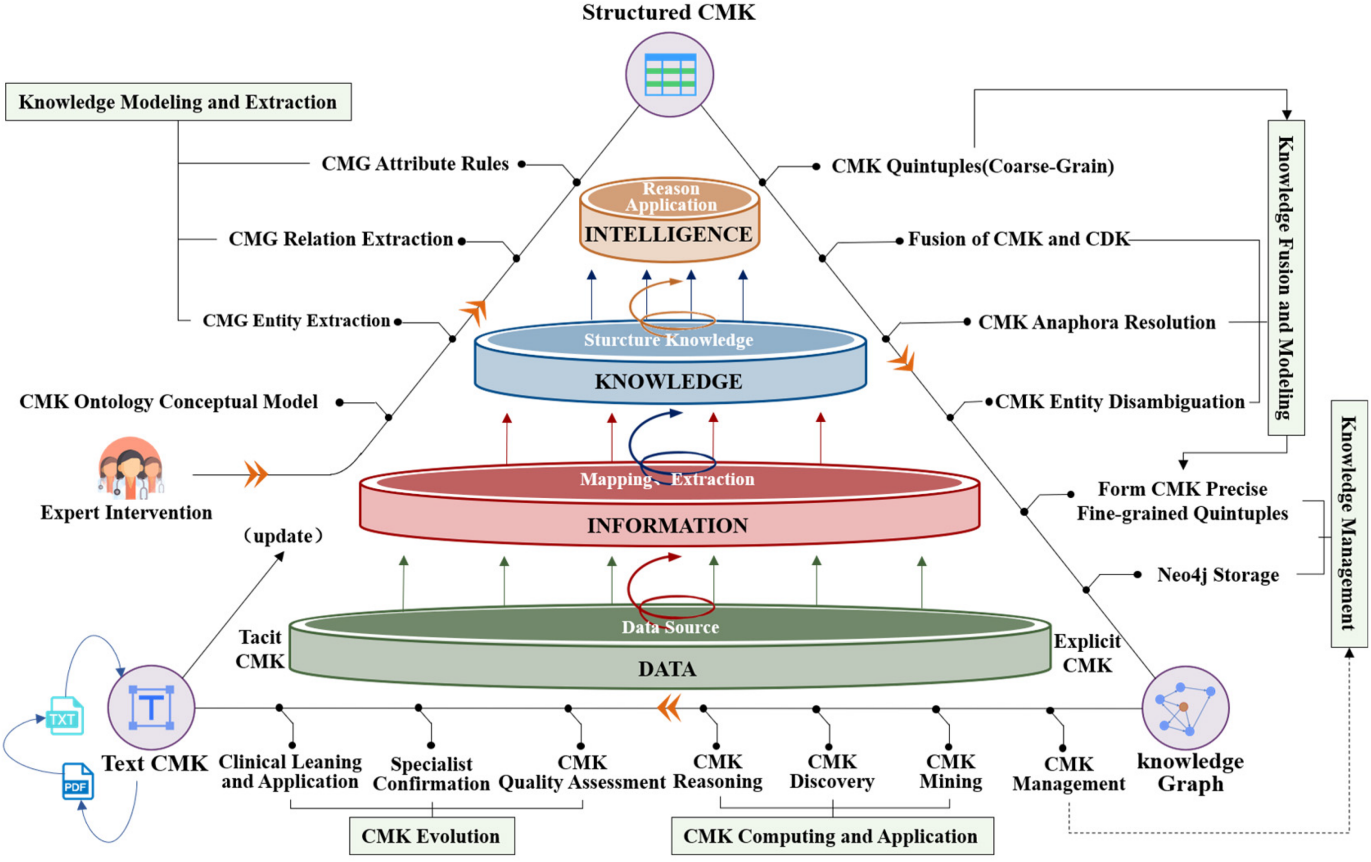
Construction of EKG-CMG and its research route in medication knowledge discovery and reasoning.

Cardiovascular research has gained increased attention in recent years, and current studies rely on data-mining techniques to extract knowledge from specific perspectives, such as diagnosis (19), medical relationship mining (20), and hypertension knowledge graph construction (21). However, the systematic acquisition of CMK remains incomplete, posing challenges to medication guidance, particularly for new clinicians. By modeling unstructured and semi-structured cardiovascular medication guidelines and consensus, this paper constructs EKG-CMG, facilitating medication knowledge reasoning and mining to enable the discovery and application. Figure 1 illustrates the technological framework for building and applying the EKG-CMG, encompassing ontology concept model design, knowledge modeling and extraction, knowledge fusion and modeling, knowledge management, knowledge computing application, and knowledge evolution.

## 2 Materials and methods

### 2.1 Overview of the research process

This study aims to construct the EKG-CMG, which can interoperate with clinicians and assist them in making medication decisions. The EKG-CMG preserves the content of cardiovascular medication knowledge in Chinese, supporting deep knowledge mining and reasoning applications. The construction process of the EKG-CMG involves steps such as knowledge preprocessing, analysis, ontology construction, extraction, fusion, and storage. The EKG-CMG has broad applications in cardiovascular, such as knowledge visualization, querying, retrieval, discovery, and reasoning, as illustrated in Figure 1.

**Figure 2:**
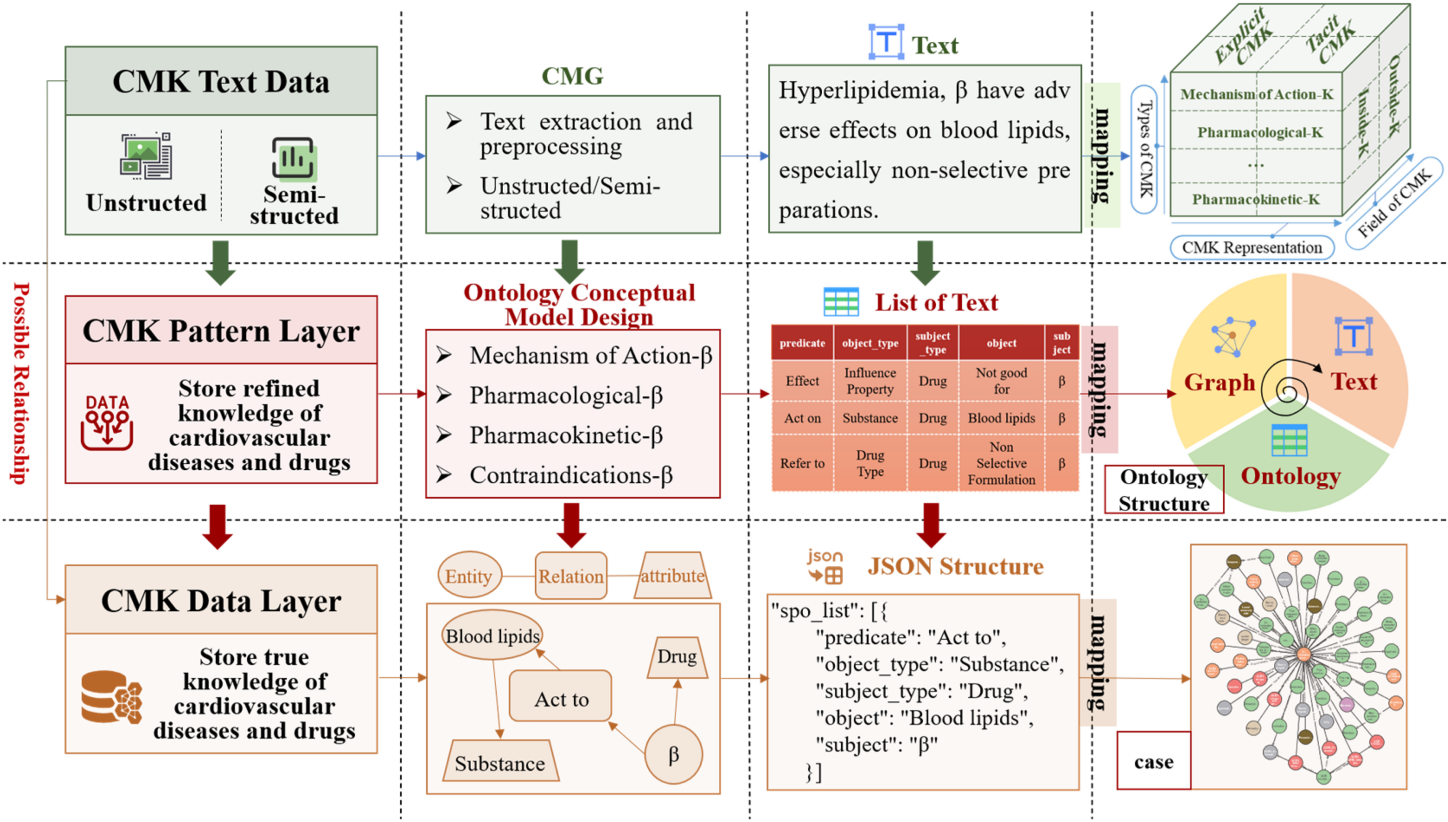
The EKG-CMG construction process diagram. (“β” denotes “β-receptor blocker” or “β-blocker”, the same below).

A core of the EKG-CMG research is the manual extraction and rigorous review of raw data by experts, ensuring the high quality, credibility, and professionalism of medication knowledge. The expert review process follows a structured methodology, including correcting medical jargon, formulating semantic forward or backward relationships, rectifying erroneous entity and relationship classifications, and eliminating inaccurate texts. Further details on this methodology are provided in Chapter 2.3. It establishes the foundation for the accurate construction and interpretation of the EKG-CMG. The EKG-CMG supports joint extraction to mine implicit knowledge and enables reasoning applications within the data. Figure 2 presents the logical process diagram of the expert knowledge graph (EKG-CMG), constructed from data sources such as cardiovascular medication guidelines and expert consensus.

### 2.2 Cardiovascular medication knowledge data sources description

The corpus of this study consists of descriptions of Chinese cardiovascular medication knowledge (CMK). Examples within the article have been translated into English to facilitate writing and dissemination. The data sources primarily encompass two main categories:

(1) **Cardiovascular medication guidelines and expert consensus**. The core data sources of the EKG-CMG include authoritative guidelines such as *Drugs for the Heart, 7th Edition*, etc. These guidelines provide detailed descriptions of the mechanisms of action, pharmacokinetics, indications, contraindications, adverse effects, complications, and drug interactions of cardiovascular drugs. Specific clinical descriptions of antithrombotic, antiplatelet, and anticoagulant drugs, fibrinolytic agents, lipid-lowering and anti-atherosclerotic drugs, as well as treatment strategies for arrhythmias, metabolic syndrome, hyperglycemia, and type 2 diabetes, constitute a highly reliable data source for EKG-CMG construction.
(2) **Expert consensus and supplementary literature** (16, 17). The EKG-CMG construction also incorporates consensus reached by experts in the cardiovascular field within China through extensive clinical practice and additional medication guideline documents. Additionally, the EKG-CMG integrates knowledge from the Cardiovascular Rational Drug Use Database (22) maintained by the National Health Science Data Center. This database encompasses detailed medication knowledge, including pharmacological effects, pharmacokinetics, indications, dosages, adverse reactions, interactions, precautions, and efficacy evaluations for 71 commonly used cardiovascular drugs. These resources substantially enhance the authority and practical utility of the EKG-CMG.

The EKG-CMG converts complex unstructured and semi-structured text into a reliable knowledge source that is intuitive, illustrated, and easy to search, enabling clinicians to access medication information efficiently. It enhances clinical comprehension and query efficiency while offering innovative techniques for in-depth knowledge exploration and discovery. The EKG-CMG provides cardiovascular clinicians with authoritative diagnostic guidance and medication references.

### 2.3 Cardiovascular medication knowledge preprocessing

#### 2.3.1 Cardiovascular medication knowledge (data) screening

**Figure 3:**
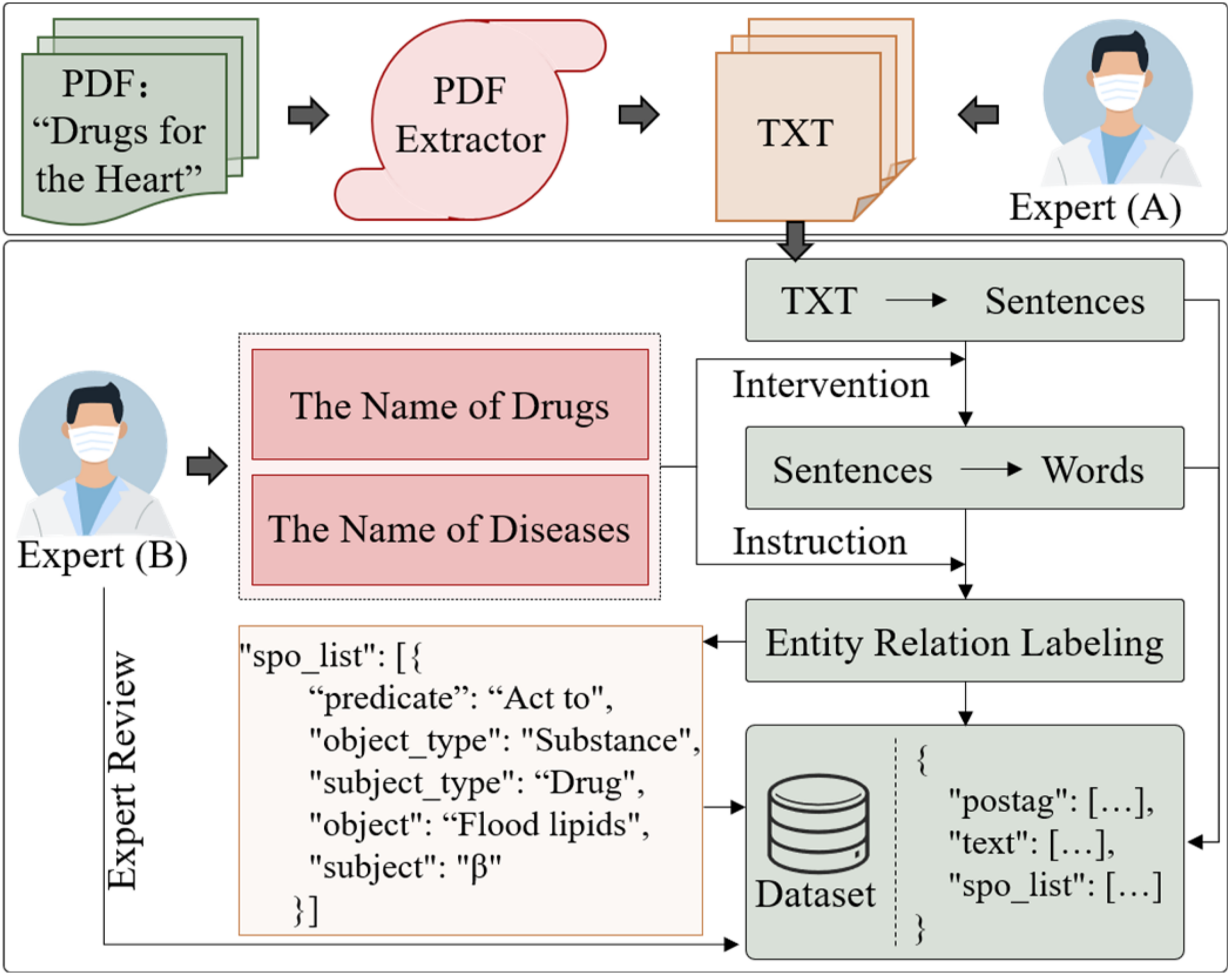
Cardiovascular medication knowledge acquisition and data preprocessing process.

The collection and cleaning of CMK involves a combination of automated text extraction, preliminary screening, and expert manual correction. The steps in Figure 3:

(1) **Text Extraction**: PDF and image text extraction tools, such as pymupdf and pdfplumber, converted the original document into plain text (txt).
(2) **Manual Filtering and Screening**: Due to specific font structures in Chinese drug names, some English characters may be recognition mistaken, such as “I” to “l”; the text serialization, table layout errors, and incomplete table entries may also impact the accuracy of medication knowledge. To address these issues, expert reviewers (A) conduct manual filtering and screening to eliminate erroneous text and ensure the accuracy of text order and content within sentences.
(3) **Expert Annotation and Quintuple Formatting**: Experts (B) annotate the medication knowledge, converting unstructured and semi-structured information into a unified, standardized quintuple, as follows:
  (1) $$\text{Y = \{ ( SUB, SU}{\text{B}}_{\text{T}}\text{, }{\text{R}}_{\text{r}}\text{, OBJ, OB}{\text{J}}_{\text{T}}\text{) | SUB }\in \text{En, OBJ}\in \text{En, }\mathrm{SU}B_{\text{T}}\in C\text{, OB}{\text{J}}_{\text{T}}\in C\text{, }{\text{R}}_{\text{r}}\in R\text{\} }$$

where En represents a set of candidate entity spans, $\text{En = \{ ( }{\text{w}}_{\text{i}}\text{, }\ldots \text{, }{\text{w}}_{\text{j}}\text{) | 1}\leq \text{i}\leq \text{j}\leq \text{n\} }$. ${\text{w}}_{\text{i}}$ and ${\text{w}}_{\text{j}}$ represent the start and end positions of the entity, respectively. “SUB” and “OBJ” represent entities, corresponding to the subject and object. $\mathrm{SU}B_{\text{T}}$ and $\text{OB}{\text{J}}_{\text{T}}$ indicate the types of these entities. The entity type set is denoted as $C=\text{\{ }{\text{T}}_{\text{1}}\text{, }{\text{T}}_{\text{2}}\text{, }\ldots \text{, }{\text{T}}_{\text{t}}\text{\} }$, and the relationship set $R=\text{\{ }{\text{R}}_{\text{1}}\text{, }{\text{R}}_{\text{2}}\text{, }\ldots \text{, }{\text{R}}_{\text{m}}\text{\} }$ represents relationships between entities, shown as “predicate” in Figure 3. This method aims to output a structured set of quintuples. Specifically, to address the overlap issue between subject and object entities, expert screening is used to identify the relationship direction within the quintuple, defined as $\text{( SUB, SU}{\text{B}}_{\text{T}}\text{, }{\text{R}}_{\text{r}}\text{, OBJ, OB}{\text{J}}_{\text{T}}\text{) }\neq \text{( OBJ, OB}{\text{J}}_{\text{T}}\text{, }{\text{R}}_{\text{r}}\text{, SUB, SU}{\text{B}}_{\text{T}}\text{) }$.

During the manual filtering process in step (2), two auxiliary dictionaries—the Cardiovascular Drug Pharmacopoeia (Supplementary Appendix Drug Pharmacopoeia) and the Cardiovascular Disease Codex (Supplementary Appendix Disease Codex)—were constructed to improve the accuracy of text information. In EKG-CMG, terms like “exertional angina pectoris” and “angina pectoris,” as well as “acute myocardial infarction” and “myocardial infarction,” are treated as distinct symptoms or diseases. As such, these expert-designed dictionaries are crucial for accurate text matching. During the part-of-speech tagging (postag) step in Figure 3, these dictionaries help prevent inaccurate word segmentation. For instance, without these dictionaries, the system might erroneously segment terms such as “Yi Chun Jie Duan” (ethanol withdrawal) into “Yi Chun,” “Jie,” and “Duan”; “Qing Guang Yan” (glaucoma) into “Qing,” “Guang,” and “Yan”; or “Xian Gan Suan Huan Hua Mei” (adenylate cyclase) into “Xian Gan,” “Suan,” “Huan Hua,” and “Mei.” Such errors can cause a significant loss of original meaning. These dictionaries enhance key-term recognition accuracy, supporting the accurate extraction of entity relationships and enabling EKG-CMG to achieve higher precision and professionalism in knowledge description. It distinguishes EKG-CMG from general medical knowledge graphs, enhancing scientific rigor and practical utility in clinical drug applications.

#### 2.3.2 Cardiovascular medication efficacy evaluation knowledge

The data sources for EKG-CMG include the Cardiovascular Medication Guidelines (CMG), expert consensus, and clinical experience. Following filtering and screening by experts (A) and annotation by experts (B), the EKG-CMG provides a foundation for precise medication knowledge and practical clinical reasoning. To this end, the study referenced authoritative expert guidance and guideline supplements, including *Drugs for the Heart* (*7th Edition*), *Chinese Guidelines for the Diagnosis and Treatment of Heart Failure 2024* (16)*, Chinese Guidelines for the Prevention and Treatment of Hypertension 2024 and etc.,* and an evaluation system for evaluating the description of CMK before and after intervention was developed, in Table 1.

**Table 1:** Cardiovascular medication efficacy evaluation knowledge Index.

Evaluation Indicators	Evaluation Description Content	Evaluation Description
Clinical symptoms	Angina pectoris, dyspnea, headache, dizziness, visual impairment, hallucinations, edema, shortness of breath, palpitations, etc.	Relieve, improve, disappear, not obvious, no recurrence, improve, subside, decrease, etc.
Physiological indicators	Blood pressure, electrocardiogram (ECG), heart rate, respiratory rate, etc.	Normalization, stability, increase, decrease, etc.
Biochemical indicators	Total cholesterol, low-density lipoprotein cholesterol (LDL-C), high-density lipoprotein cholesterol (HDL-C), triglycerides, and proteinuria, etc.	Decrease, reduce, control, improve, etc.
Functional indicators	Ejection fraction (EF), echocardiography results, etc.	Improve, recover, stabilize, optimize, etc.
Biomarker levels	C-reactive protein (CRP), troponin, cyclic adenosine monophosphate (cAMP), etc.	Decline, normalize, recover, stabilize, etc.
Event rate	Mortality, recurrence, long-term survival, etc.	Decline, decrease, no change, etc.

#### 2.3.3 Cardiovascular medication knowledge standardization

The data sources for EKG-CMG include consensus medication knowledge accumulated by domestic cardiovascular experts in clinical practice, supplementary guidelines, medication data from the National Health Science Data Center, et al. (22). To avoid issues such as non-standard medication terminology and concept ambiguity, the EKG-CMG employs a standardized processing method, referencing terminology standards from *Drugs for the Heart* (*7th Edition*). This approach enhances both the quality of medication knowledge and the professionalism of EKG-CMG. As shown in Table 2, the standardized processing method includes term classification for medication knowledge, synonym unification, consistency in drug names and aliases, inclusion of reference terms, and streamlined descriptions for redundant terminology.

**Table 2:** Example of data standardization.

No.	Standardized methods	Original terminology	Standardized terminology
1	Unification of term classification categories	Short-acting nitrates, long-acting nitrates	nitrate drugs
2	Unification of synonyms	ACE inhibitors, angiotensin-converting enzyme inhibitors	ACEI
3	Normalization of irregular expressions	Research status of angina pectoris prognosis	Angina pectoris prognosis study
4	Filling in reference terms	Intravenous beta-blockers or verapamil	Intravenous beta-blockers, intravenous verapamil
5	Simplification of redundant terms	Complications related to hypertension	Hypertension complications

### 2.4 Construction of cardiovascular medication knowledge ontology

**Figure 4:**
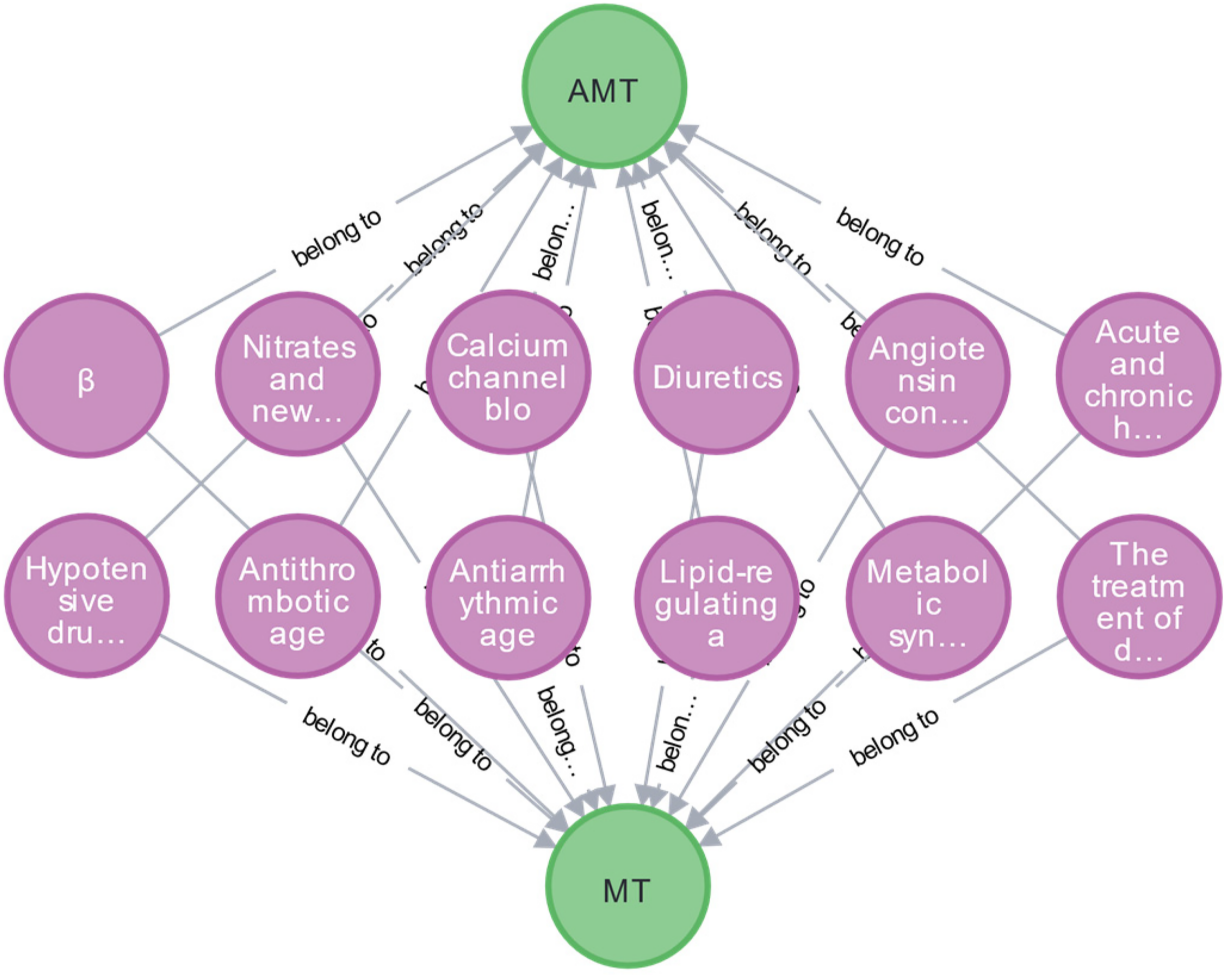
The construction of the MT and AMT ontology concept. (The green nodes are the attribute category ontology of medication types, including MT and AMT, and the purple nodes are the MT ontology, including beta-blockers, nitrates, and new anti-angina drugs, etc.

**Figure 5:**
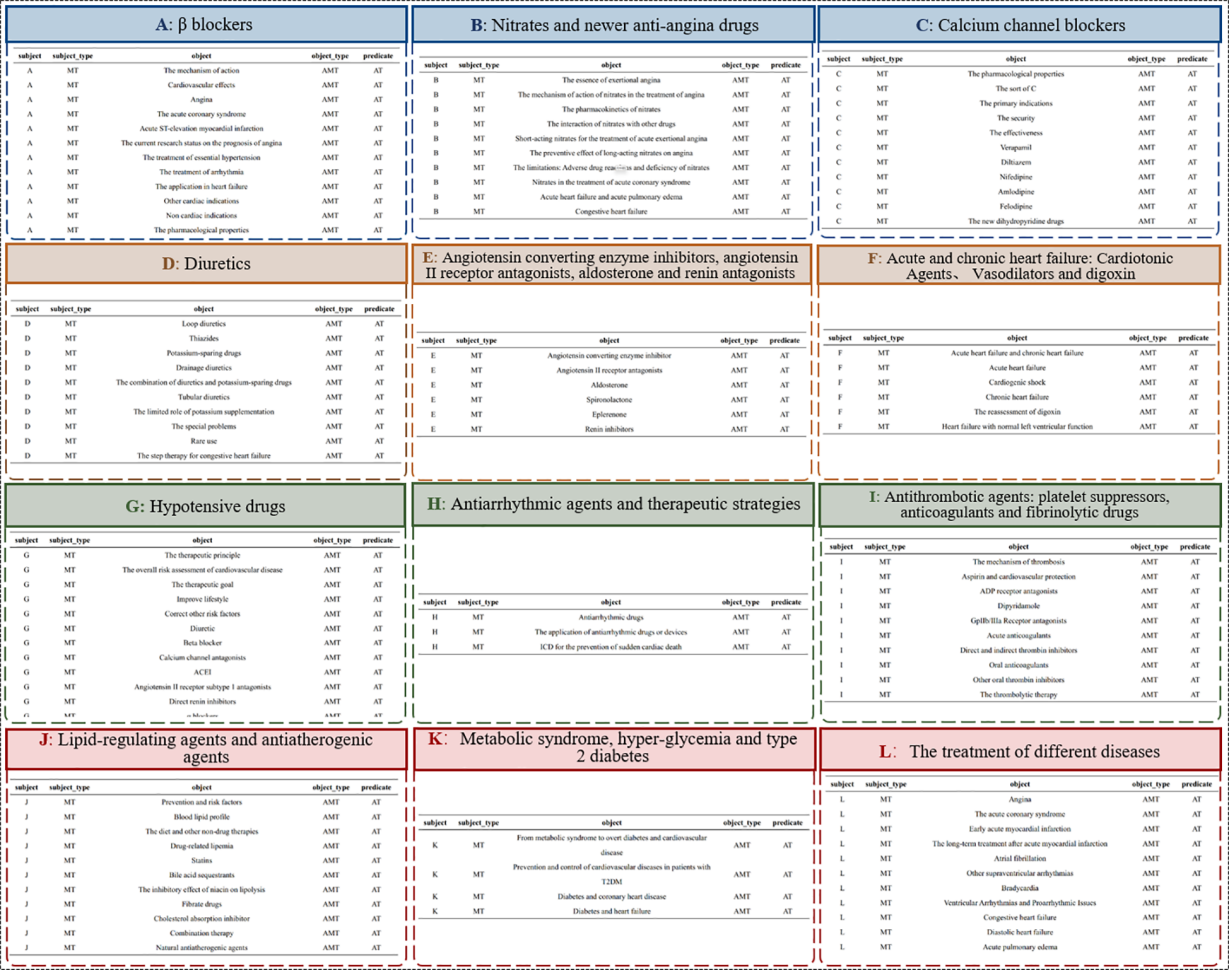
The construction of MT and AMT ontology (A∼L represents MT ontology categories for different drug types).

To refine entities and relationships within medication knowledge, the EKG-CMG defines a medication ontology, termed the Medication Type (MT). It includes categories like beta-blockers, nitrates, new anti-anginal drugs, calcium channel blockers, diuretics, antithrombotic drugs, platelet inhibitors, anticoagulants, fibrinolytic drugs, and lipid-regulating or anti-atherosclerotic drugs, as illustrated in Figure 4. Together with specific drug names (e.g., atorvastatin, aspirin), the EKG-CMG defines the ontology attributes as Attributes of Medication Type (AMT), encompassing drug mechanism of action, pharmacokinetic and pharmacological properties, indications, contraindications, adverse reactions, and complications. Additionally, medication attributes include dosage forms, frequency, cycle, and administration method (route), with specific examples such as tablets, three times daily, oral administration, and intravenous injection. Symptom attributes, such as complications and comorbidities, also include manifestations like hypertension, headache, and angina, as illustrated in Figure 5.

Additionally, we have clarified the full names of previously truncated nodes, such as: “Nitrates and new…” → “Nitrates and newer anti-angina drugs.” “Angiotensin con…” → “Angiotensin converting enzyme inhibitors, angiotensin II receptor antagonists, aldosterone, and renin antagonists.” “Acute and chronic h…” → “Acute and chronic heart failure: positive inotropic agents and vasodilators are known to be dissolute.” “Hypotensive dru…” → “Hypotensive drugs.” “Metabolic syn…” → “Metabolic syndrome, hyperglycemia, and type 2 diabetes.” “The treatment of d…” → “The treatment of different diseases.” The relationships in the figure are all labeled as “Belong to.”

The EKG-CMG has established a multidimensional relational data mining model, referred to as ontology relations. These relations include “disease-disease,” “drug-AMT,” “patient-AMT,” “drug combination-drug,” “drug effect-AMT,” “drug effect-drug characteristics,” “drug effect-drug,” “drug category-AMT,” “MT-AMT,” “symptom-AMT,” “treatment method-AMT,” “treatment method-symptom,” “disease-disease stage,” and others. For example, using beta-blockers as a case study, multidimensional relationships were mined, revealing 388 associated relationship descriptions. These include type attributes (Attributes of Type, AT), indications, combined medications, treatment methods, stage relations, and others, in Table 3. Fine-grained relational data significantly enhances EKG-CMG’s capability for detailed knowledge representation.

**Table 3:** Sample relational descriptions obtained from knowledge mining (beta-blockers as an example).

No.	Entity/ontology category	Relationship
1	Disease-disease	Contain
2	Drug-AMT	Type attribute (AT), drug relationship, combined use, combined effect is better than, etc.
3	Patient-AMT	Add, use, give, contraindications to, composition, initial treatment, beneficial, intolerable, try to give, standard treatment, etc.
4	Drug combination-drug	Combination medication, include, drug combination, etc.
5	Drug effect-AMT	Description, drug relationship, efficacy comparison, adverse reactions, effect, application strategy, etc.
6	Drug effect-drug characteristics	Drug comparison, efficacy comparison, etc.
7	Drug effect-drug	Drug relationship, should, etc.
8	Drug category-AMT	Interaction exists, acts, is not, belongs to, etc.
9	MT-AMT	Type attribute (AT), etc.
10	Symptom-AMT	Relieve, affect, aggravate, cannot effectively relieve, indication, inhibit, lower blood pressure, cause, cause, may aggravate, application description, etc.
11	Treatment method-AMT	Treatment, use with caution, application, application description, indication, etc.
12	Treatment method-symptom	Is, relieve, improve, etc.
13	Disease-disease stage	Stage, etc.

The EKG-CMG comprises an ontology module and a data module. The ontology module defines the theoretical foundation of the EKG-CMG, hierarchically structuring medication concepts through a logical framework to provide an overarching structure for the knowledge graph. The data module encompasses knowledge content related to cardiovascular medication, serving as an instantiation of the ontology module. The primary objective of ontology construction (23) is to obtain, describe, and systematically express cardiovascular medication knowledge (CMK). The ontology of the EKG-CMG is founded on widely accepted terms and concepts acknowledged by experts in medication knowledge. Concepts and terms are organized via ontology logical relationships that can fully present the content and internal logical relationships of CMK.

The EKG-CMG construction employs two technical approaches: top-down (24) and bottom-up (25), which complement each other in building the knowledge graph. The top-down approach begins with cardiovascular medication concepts, organizing their logical relationships to create a hierarchical structure within the ontology module. Data modules containing medication knowledge (e.g., entities and instances) are mapped to the ontology module to increase the specificity and accuracy of the medication ontology. Conversely, the bottom-up approach extracts entity and relationship knowledge from CMG, integrates it into the EKG-CMG, and continuously iterates on and updates the ontology module based on graph content, enhancing its applicability. This iterative process facilitates dynamic evolution within the ontology. This study prioritizes the top-down approach to ensure the EKG-CMG clinical guidance, professionalism, accuracy, and stability.

**Figure 6:**
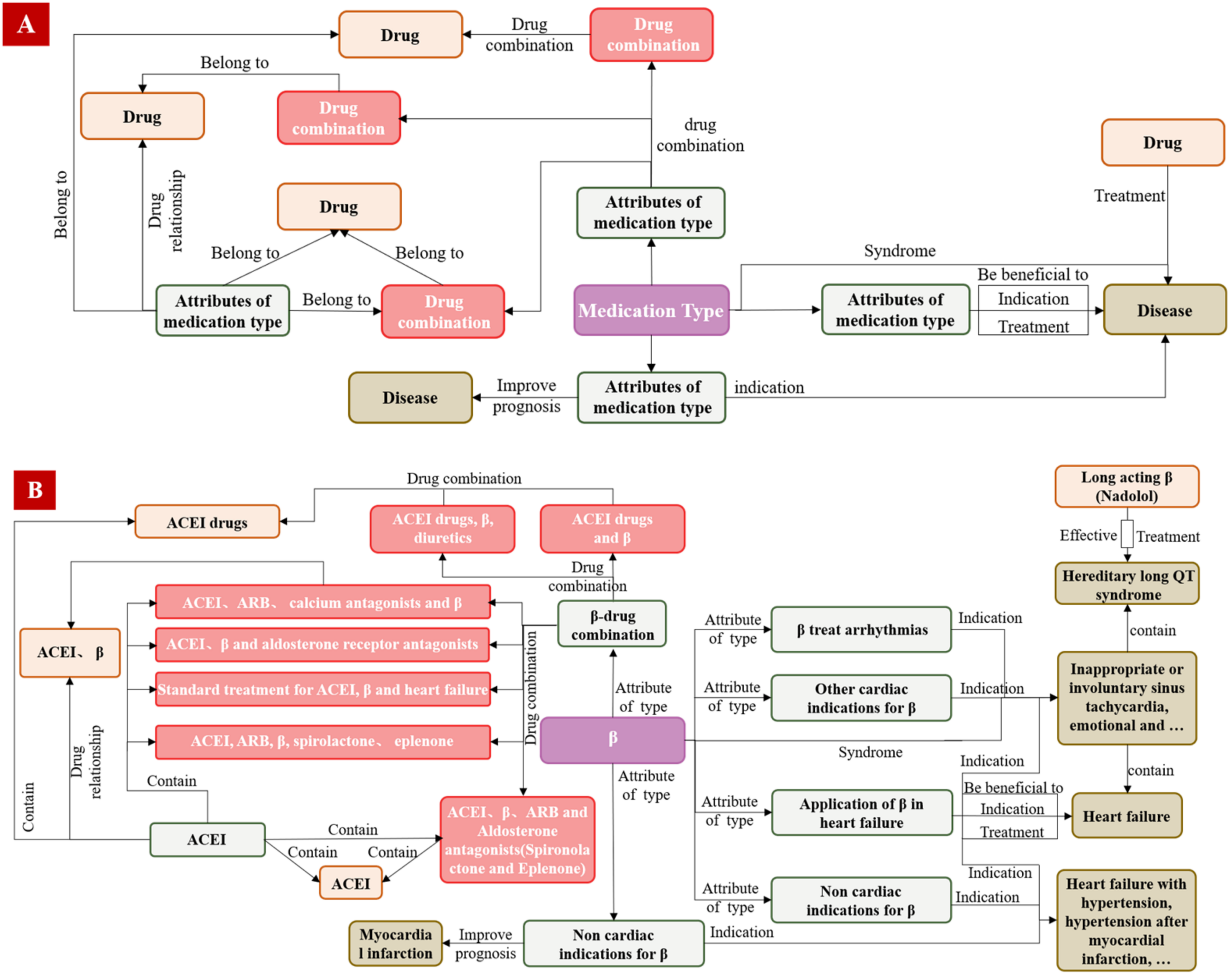
The logical relationship framework diagram between the partial ontology module **(A)** and the partial data module **(B)**.

During the expert screening process, CMK was initially organized and standardized, and they defined the ontology module, leveraging existing medication concepts and semantic relationships. The data module models medication entities and relationships using a quintuple format, forming expressions like “entity-entity type-relationship (predicate)-entity-entity type” and “entity-entity type-attribute relationship-attribute-attribute value” as shown in Formula 1. The quintuple design facilitates the mapping of information and knowledge to concepts and relationships within the ontology. Each “medication type” (MT) has its own distinct data module. For instance, Figure 6 illustrates the logical connection between the data and ontology modules for β-receptor blockers, highlighting high-frequency relationships.

### 2.5 Cardiovascular medication knowledge extraction

The data sources of the EKG-CMG encompass both unstructured data, such as CMG and related medication consensus, and semi-structured information on certain cardiovascular medications provided by the National Health Data Center (22). Given the complex structure and varied formats of the CMK, discrepancies in descriptions appear across some consensuses and guidelines. Although named entity recognition (NER) methods are widely used to extract entities and relationships, they encounter significant challenges when extracting complex knowledge from specialized cardiovascular texts, particularly regarding the recognition of professional cardiovascular terms.

**Figure 7:**
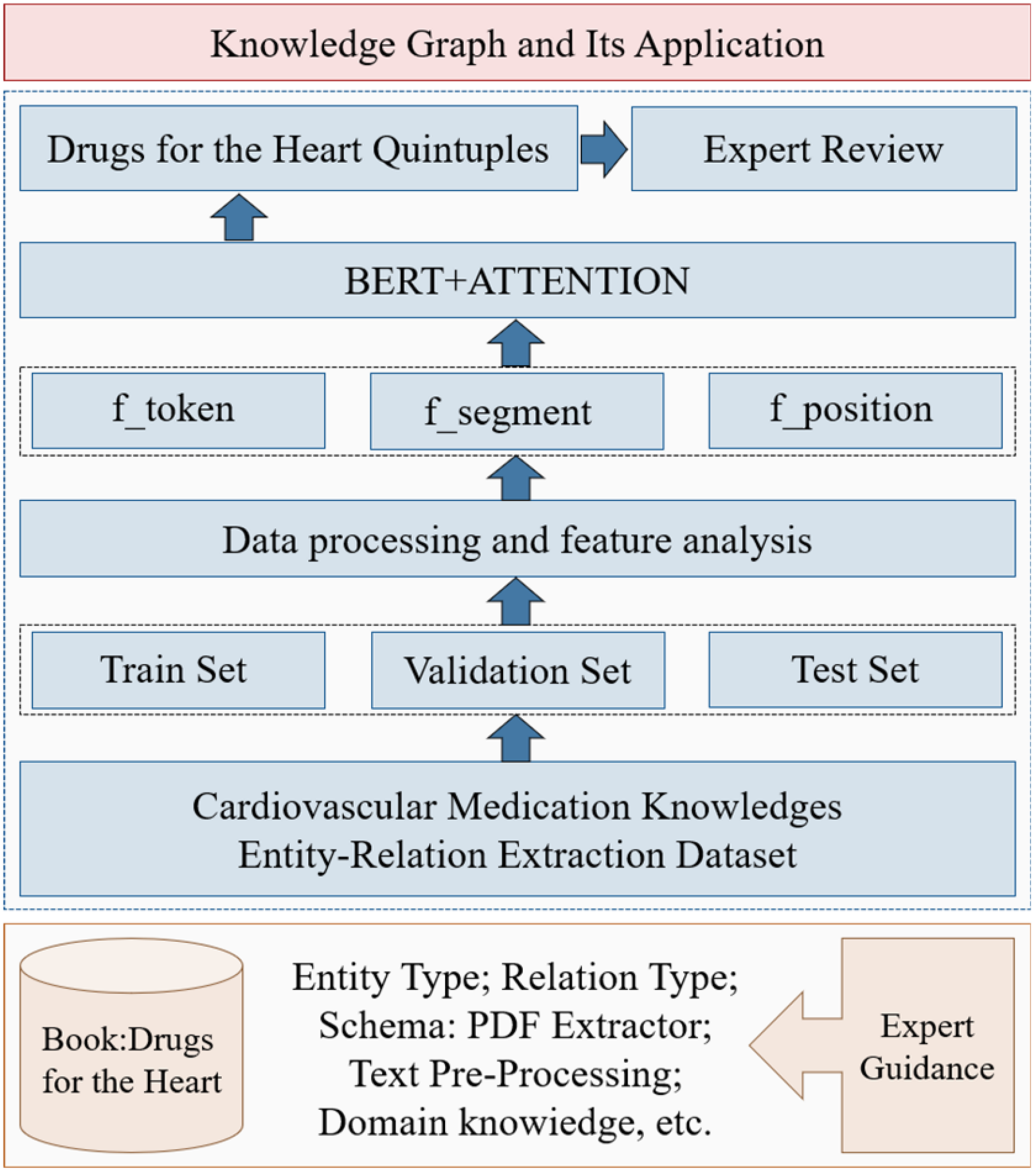
Our proposed model for joint extraction of quintuple information of entities and relations.

To address these challenges, we constructed a pharmacopeia and disease codex for CMK and developed an entity-relationship quintuple extraction model to minimize discrepancies between CMK semantics and actual meaning. In the EKG-CMG, accurately extracting dictionary-assisted entities enhances sentence semantic expression. It leads to a higher correlation between entity features and relationship information and greater semantic accuracy. This study treats entities as explicit features of sentences and relationships as implicit semantic features, employing BERT and Attention Mechanisms to construct a joint model for entity-relation extraction. Special keywords, such as those from the disease codex and pharmacopeia, assist in extracting quintuple information, and refining and optimizing relationship classification research. Consequently, the quintuple information extraction model enhances the accurate extraction and expression of complex entities and relationships in the EKG-CMG. The extraction model’s structural design is in Figure 7.

### 2.6 Cardiovascular medication knowledge storage

**Figure 8:**
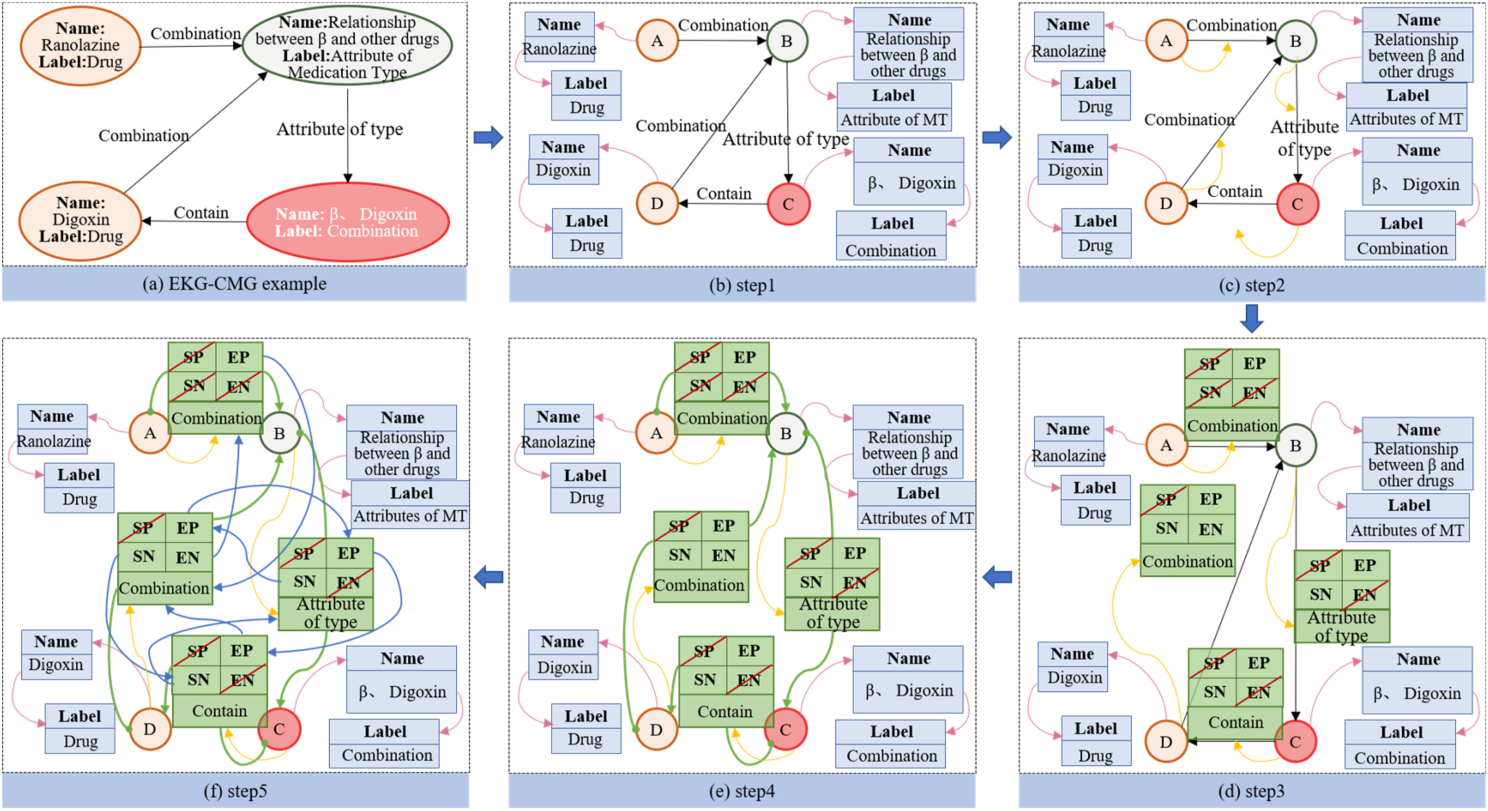
Construction principle of the EKG-CMG using Neo4j graph.

The EKG-CMG utilizes the Neo4j graph database (26, 27) to store graph-structured CMK. Neo4j organizes CMK in a graph format with nodes, edges, and attributes, and is currently one of the most widely used high-performance NoSQL graph databases (28). The high availability, stability, scalability, and intuitive visualization features of Neo4j make it an ideal storage solution for constructing EKG-CMG. Figure 8 illustrates the principle of storing knowledge on beta-blockers in Neo4j.

The Figure 8 illustrates an example using beta-blockers. Part (a) shows the attribute graph of EKG-CMG, while part (b) presents the attribute deconstruction graph, with the pink arrow in Step 1 pointing to the deconstructed attribute. Part (c) depicts the deconstruction graph of the point, the object structure of the point is established; in step 2, the yellow arrow points to the first edge connected to the node. Part (d) shows the object structure of the mapped edge. where, in step 3, SP and SN represent the previous and next edges of the starting point, and EP and EN represent those of the endpoint. Part (e) indicates the direction of the starting and end points in the object structure of the complete edge. In step 4, the solid circle on the green line represents the starting point, while the arrow end represents the endpoint. Part (f) illustrates the object structure of the completed non-empty edge, with the blue arrow in step 5 indicating a non-empty edge.

**Figure 9:**
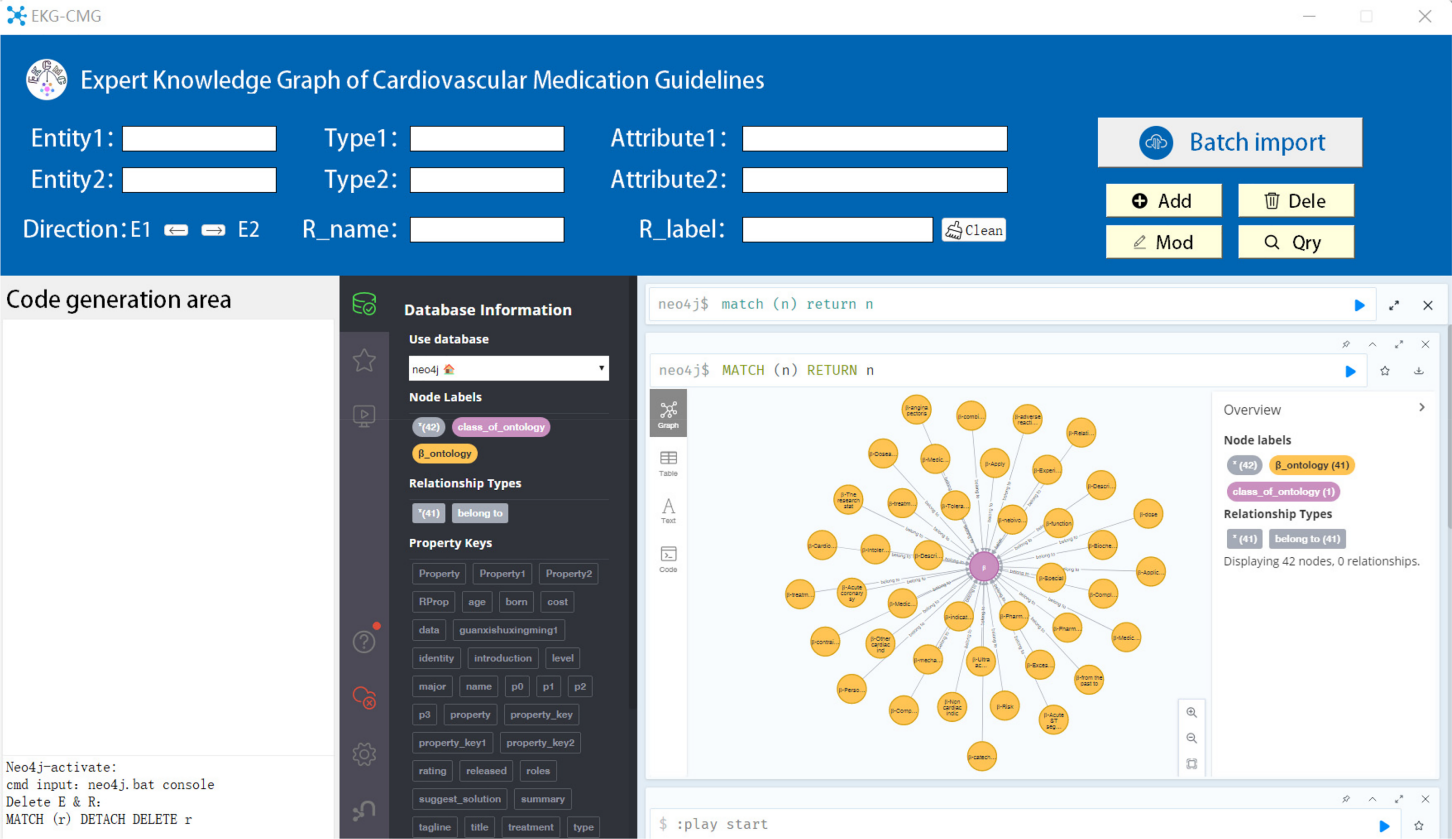
The system interface diagram for automatically storing entity relationships of CMK into Neo4j and generating knowledge graphs (*Entity1* is the name of entity 1, *Type1* is the category of entity 1, *Attribute1* is the attribute of entity 1. The same applies to entity 2. *Direction* refers to the direction of the relationship, *R_name* is the relationship name, and *R_label* is the relationship category).

During the construction of EKG-CMG, an autonomous knowledge graph generation system for cardiovascular drug knowledge was developed using Python and the Tkinter GUI programming framework. This system automatically generates programmable Cypher code. After importing data, EKG-CMG iteratively processes each record to generate Cypher code for knowledge graph nodes and edges, calls and executes Neo4j commands, and efficiently supports the addition, deletion, modification, and querying of graph nodes, edges, and their attributes, as shown in Figure 9. EKG-CMG’s automated processing flow not only enhances the efficiency of constructing the medication knowledge graph but also ensures data management accuracy, convenience, and scalability.

## 3 Results

### 3.1 Cardiovascular medication knowledge ontology concepts and types

**Figure 10:**
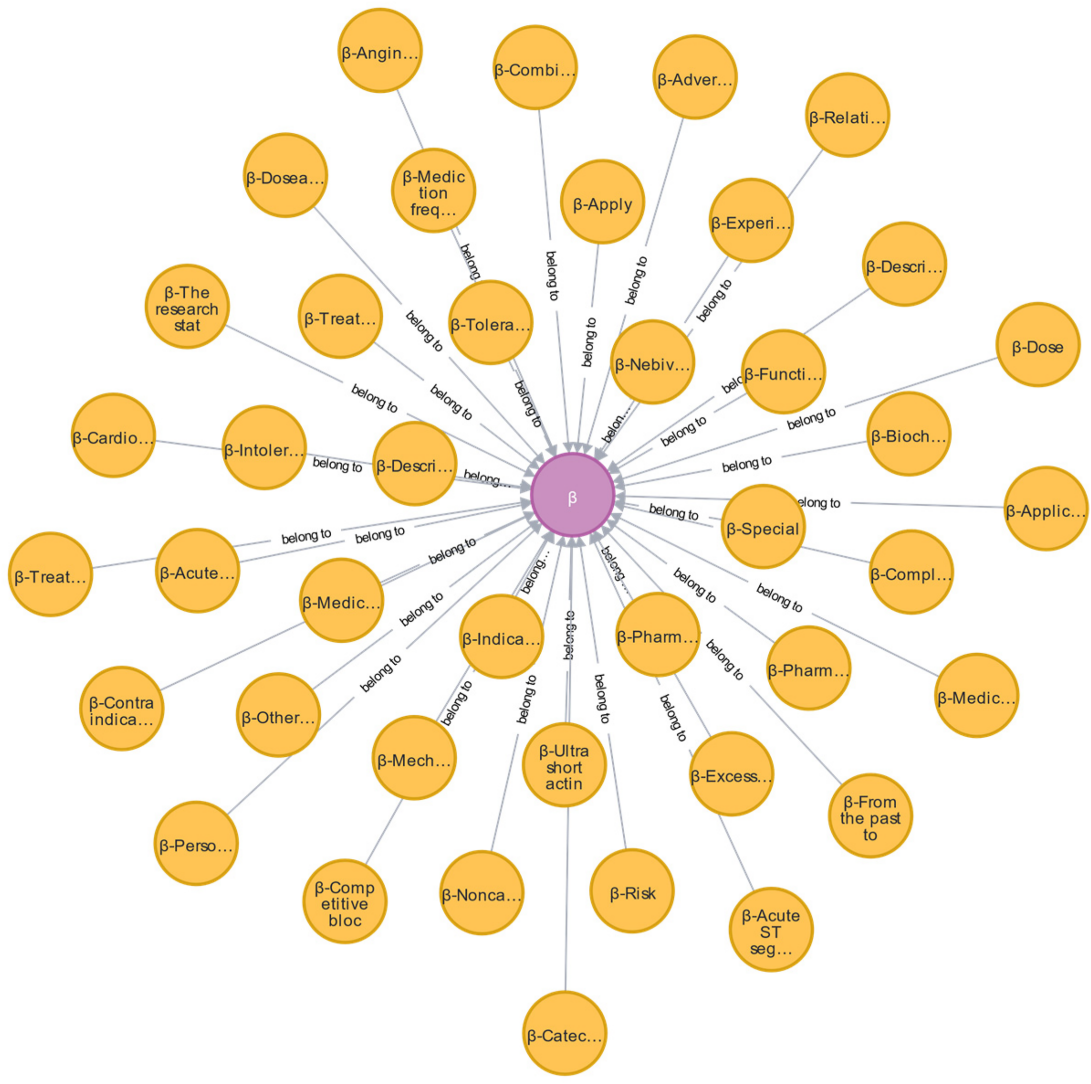
Ontology concept map of β-blockers (purple nodes are β-blocker ontology, yellow nodes are β-blocker AMT ontology, and edges are β-blocker relationship attribute ontology. Additionally, we have provided a reference to Table 4, where the full content of the entities is listed. The relationships in the figure are all labeled as “Belong to.”).

During the development of the EKG-CMG, CMG, expert consensus, and frontline clinical experience are utilized to create a highly reliable and accurate knowledge system for drug use, with knowledge logic visualized in a knowledge graph format. For example, in the case of beta-blockers, 17 conceptual terms across 41 ontologies have been defined, as shown in Figure 10 and Table 4. These conceptual terms include drugs, drug combinations, diseases, patients, combined medications, behaviors, treatment methods, research, drug applications, complications, contraindications, reports, drug effects, indications, symptoms, drug contraindications, and adverse reactions.

**Table 4:** Concept ontology terms for the type of use of beta-blockers.

No.	AMT ontology of β blockers	Concept terms	No.	AMT ontology of β blockers	Concept terms
1	β-Special beta blockers	Drug	22	β-Apply	Drug applications
2	β-Ultra short acting intravenous	Drug	23	β-Route of administration	Drug applications
3	β-Nebivolol	Drug	24	β-Medication cycle	Drug applications
4	β-Other drug relationships	Drug combinations	25	β-Medication frequency	Drug applications
5	β-Angina pectoris	Diseases	26	β-Complications and selections	Complication
6	β-Acute ST segment elevation myocardial infarction	Diseases	27	β-Contra indications	Contraindications
7	β-Personnel	Patients	28	β-From the past to the future	Report
8	β-Combination medication	Combination medications	29	β-Biochemical substances	Report
9	β-Catecholaminergic	Combination medications	30	β-Cardiovascular effects	Drug effects
10	β-Mechanism of action	Behaviors	31	β-Description of resistance to beta blockers	Drug effects
11	β-Pharmacological properties	Behaviors	32	β-Intolerance	Drug effects
12	β-Pharmacokinetic characteristics	Behaviors	33	β-Tolerance	Drug effects
13	β-Function	Behaviors	34	β-Competitive blocking effect	Drug effects
14	β-Treatment of primary hypertension	Treatment methods	35	β-Indications	Indications
15	β-Treatment of arrhythmia	Treatment methods	36	β-Acute coronary syndrome	Symptom
16	β-The research status of the prognosis of angina pectoris with β	Research	37	β-Other cardiac indications	Symptom
17	β-Description	Research	38	β-Noncardiac indications	Symptom
18	β-Experimental study description	Research	39	β-Excessive use	Symptom
19	β-Application in heart failure	Drug applications	40	β-Risk	Drug contraindications
20	β-Doseage of medication	Drug applications	41	β-Adverse reactions	Adverse reactions
21	β-Dose	Drug applications			

Additionally, Table 5 systematically organizes information sources for drugs, drug combinations, diseases, patients, and combined medications from guidelines, expert consensus, standards, and clinical plans, supplementing these ontologies with expert clinical experience and real outpatient data.

**Table 5:** Meaning and origin of the ontological concepts of cardiovascular medication knowledge.

No.	Concept terms	Meaning	G	EC	St	Sc	R	EE
1	Drugs	Chemical substances or biological products used to prevent, treat or diagnose cardiovascular diseases. Acting on the human body through specific physiological or biochemical pathways, changing the course of the disease or symptoms.	✓	✓	✓	✓		
2	Drug combinations	A combination of two or more cardiovascular drugs, such as ACEI drugs, which are antihypertensive drugs with “pril” at the end of the drug name. The elements in the combination include captopril, enalapril, etc.	✓	✓	✓	✓		
3	Diseases	A physiological or pathological state manifested as abnormality in the function or structure of the body.	✓	✓	✓	✓		
4	Patients	An individual who is receiving treatment for a disease state.	✓					
5	Combination medications	Combination therapy using multiple drugs or treatment methods. Combination therapy can enhance the therapeutic effect or reduce drug resistance and side effects.	✓	✓	✓	✓		
6	Behaviors	Describe the specific physiological or biochemical effects of cardiovascular drugs in the body.	✓	✓	✓	✓		
7	Therapeutic approaches	Medical measures taken to improve or cure disease symptoms, including drug therapy, coronary artery bypass grafting, magnetorheological blood pressure reduction, etc.	✓	✓	✓	✓		✓
8	Research	Systematic discussion of drug effects, efficacy and safety through experiments, clinical trials or observational studies to provide scientific basis for indications and contraindications of cardiovascular drugs.	✓		✓	✓		
9	Drug applications	How the drug is actually used in clinical practice, including dosage form, dosage, and usage, etc.	✓	✓	✓	✓		✓
10	Complications	Diseases may be complicated by other diseases during their course, and complications are more complicated to treat and require careful handling.	✓	✓	✓	✓		
11	Contraindications	Describe the medical rationale for why a drug should not be used under certain conditions.	✓	✓	✓	✓		
12	Reports	Literature or monitoring data on drug use, adverse reactions, biochemicals, or efficacy.	✓		✓	✓		
13	Drug effects	Clinical outcomes or effects of drug treatment, such as efficacy, incidence of complications of heart failure, and survival of stroke patients.	✓	✓	✓	✓	✓	✓
14	Indications	Refers to the scope and standards for the application of cardiovascular drugs, cardiovascular surgery and other methods.	✓	✓	✓	✓		
15	Symptoms	Subjective sensations or clinical manifestations of patients due to diseases, such as chest tightness, palpitations, and panic, are often used to evaluate the effectiveness of treatment.	✓	✓	✓	✓	✓	✓
16	Drug contraindications	Precautions that must be followed when using specific drugs to avoid possible side effects or adverse reactions.	✓	✓	✓	✓	✓	
17	Adverse reactions	Unexpected or harmful reactions caused by drugs when used at normal doses.	✓	✓	✓	✓	✓	

G, guideline; EC, expert consensus; St, standards; Sc, schemes; R, real outpatient data; EE, expert clinical practice experience.

### 3.2 Semantic relations of cardiovascular medication knowledge concepts

Using beta-blockers as an example, 17 types of semantic relationships (ontologies/entity attributes) in the medication concept have been defined, including inclusion, description, indication, equivalent description, AT, combined medication, treatment, action, influence, drug combination, contraindications, attributes, cause, improvement, display, and stage. These relationships are derived from guidelines, expert consensus, and real outpatient data analysis, with Table 6 providing an organized description of their meanings and sources.

**Table 6:** Connotation and origin of CMK semantic relations.

No.	Relationships	Meaning	G	EC	R
1	Contains	Indicates the sub-items or elements contained in a category, emphasizing the collection relationship.	✓	✓	
2	Description	Describes a more detailed description of drug characteristics, mechanism of action, effect, dosage, and treatment strategy.	✓	✓	
3	Indications (Drugs)	Describes the clinical basis for the application of drugs, treatment methods, or medical operations to specific diseases or conditions, which is a subset of indications.	✓	✓	
4	Equivalent description	Describes different expressions or terms for the same drug or treatment plan.	✓	✓	✓
5	AT	Describes the category characteristics of drugs or treatments.	✓	✓	
6	Combination medication	Describes the strategy of using multiple drugs in treatment.	✓	✓	
7	Treatment	Describes the relationship between medical interventions for specific conditions.	✓	✓	
8	Action	Describe the specific physiological or biochemical effects of a drug in the body.	✓	✓	✓
9	Effects	Describe the intervention of a drug or treatment on body functions or specific pathological conditions.	✓	✓	✓
10	Indications	Describe the specific conditions under which a drug or treatment is actually needed for a specific disease, sign or symptom.	✓	✓	
11	Drug combination	Describe the set of drugs and the individual drugs they contain when taking drugs together.	✓	✓	
12	Contraindications	Describe the medical reasons why a drug or treatment should not be used under specific conditions.	✓	✓	
13	Properties	Describe the intrinsic characteristics or quantitative indicators of a medical entity, such as the attributes of a drug entity can be pharmacological effects and dosage.	✓	✓	✓
14	Causes	Describe the adverse reactions or side effects that a drug may cause.	✓	✓	✓
15	Improves	Describe the effect of a drug on the symptoms of a disease, including “can improve”, “improve prognosis” and “cannot improve” in this study.	✓	✓	✓
16	Shows	Describe the scientific results shown by research of medication knowledge.	✓	✓	
17	Stage	Describe different stages/periods of the same disease.	✓	✓	

G, guideline; EC, expert consensus; R, real outpatient data.

**Figure 11:**
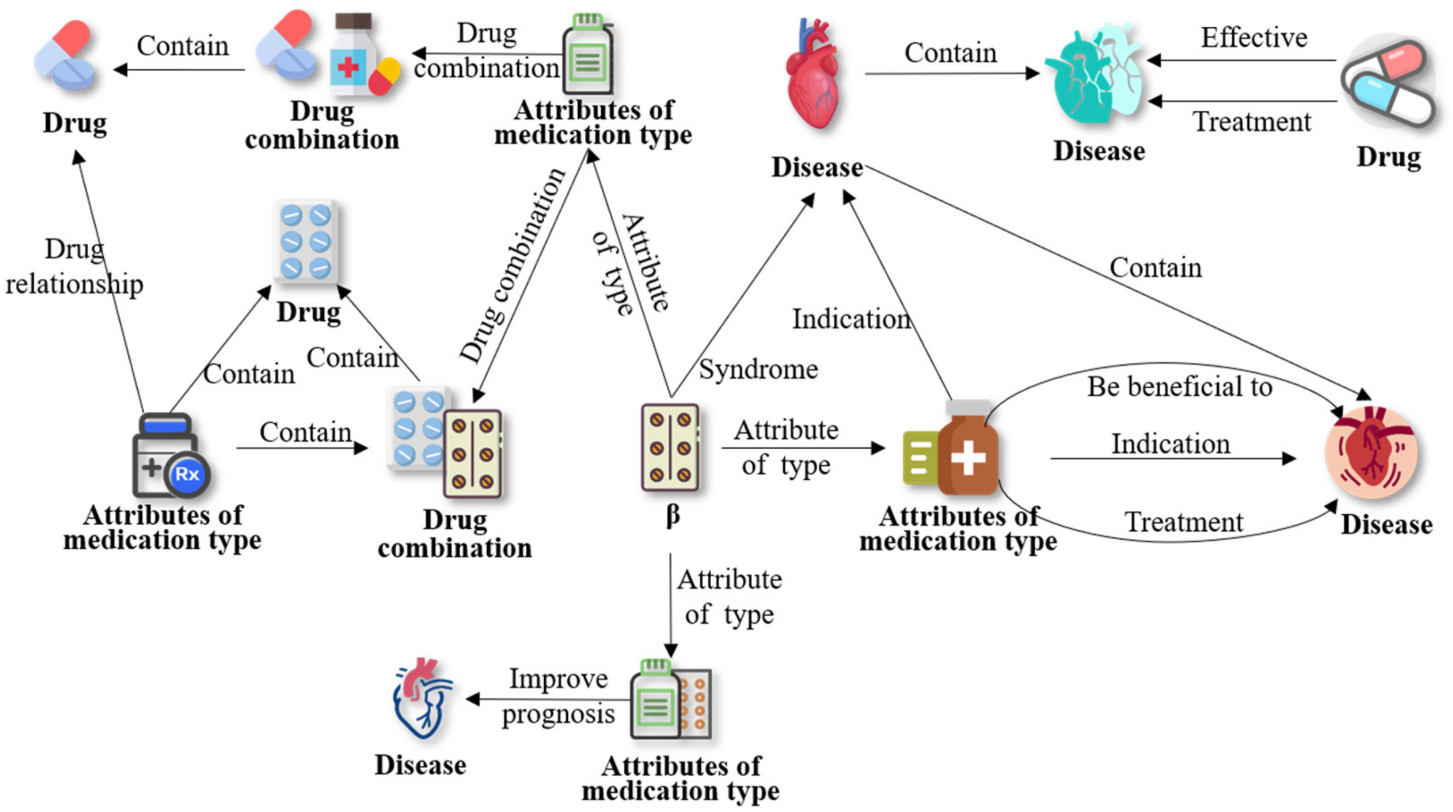
Ontological concepts and relationship framework diagram of beta-blockers in EKG-CMG.

Our prominent relationships are weighted and graded based on the frequency of two-dimensional relationships between entities, to convey the strength of relationships between medication ontologies/entities and provide readers with a more accurate understanding of CMK. The Figure 11 illustrates an example of ontology concepts and relationships related to beta-blockers. This ontology framework can serve as a general model for cardiovascular medication concepts.

### 3.3 Expert knowledge graph of cardiovascular medication guidelines knowledge

This study extracted key information from unstructured and semi-structured texts, quantitatively assessing the significance and weights of relationships based on frequency statistics, with higher weights assigned to frequently occurring relationships. All extracted knowledge results underwent strict standardization and expert evaluation to identify and rectify potential errors and deficiencies. Additionally, the extracted content has been stored in Neo4j and thoroughly reviewed by cardiovascular experts to ensure the rationality and accuracy of the findings from a professional perspective. Finally, using beta-blockers as a case example in EKG-CMG, Neo4j was employed to construct a medication knowledge graph containing 1,197 nodes and 1,351 relationships, as shown in Supplementary Figure S12 (Supplementary Appendix 1: Medication knowledge graph in the EKG-CMG using beta-blockers as an example.), establishing a foundational structure for the structured presentation of CMK.

**Figure 12:**
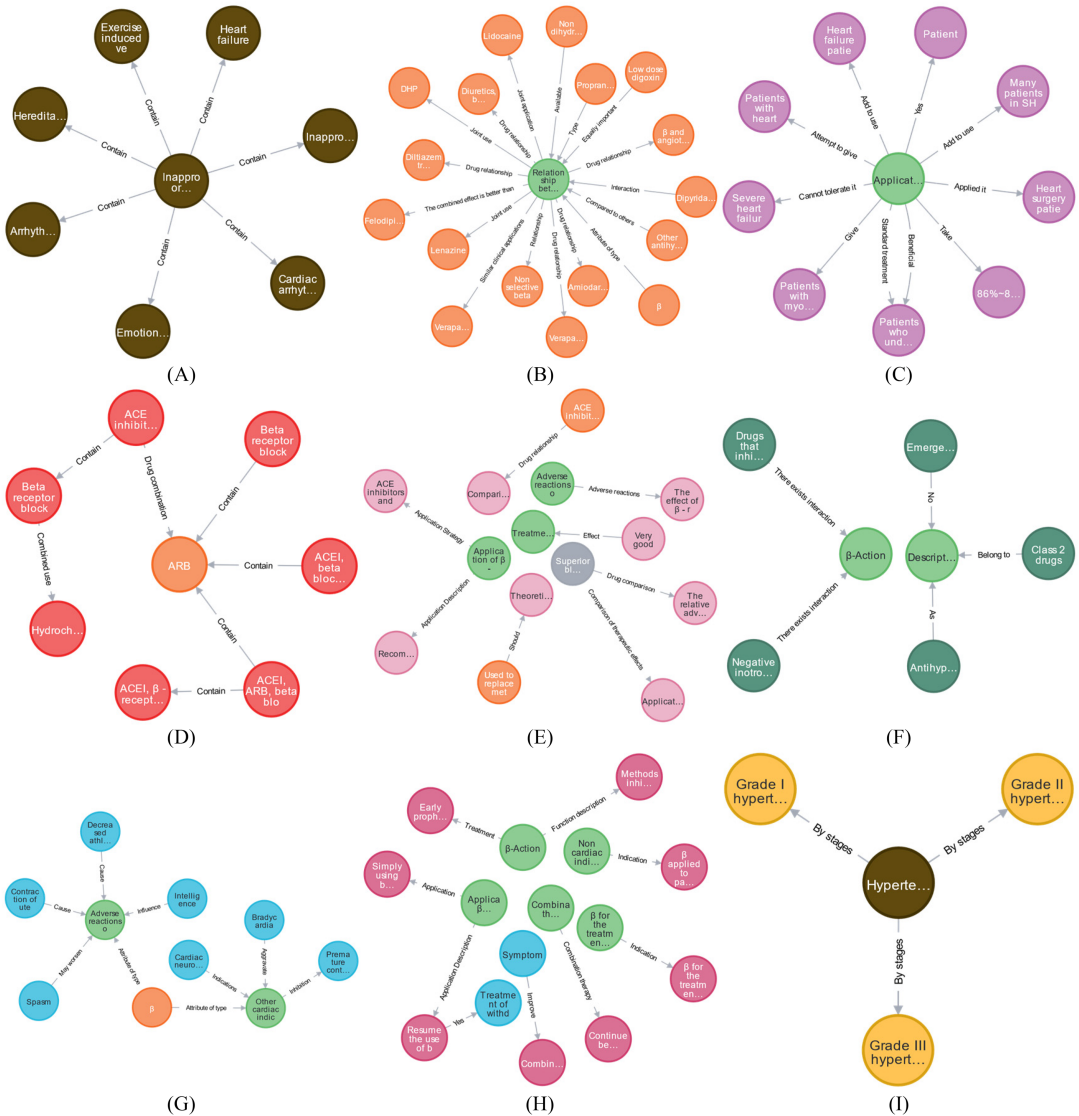
Presents the concept and relationship distribution within EKG-CMG. **(A)** Disease-disease. **(B)** Drug-AMT. **(C)** Patient-AMT. **(D)** Drug combination-drug. **(E)** Drug effect-AMT, drug effect-drug characteristics, and drug effect-drug relationships. **(F)** Drug category-AMT. **(G)** MT-AMT and symptom-AMT relationships. **(H)** Treatment method-AMT and treatment method-symptom relationships. **(I)** Disease-disease stage. (In this figure, nodes represent entities, edges represent relationships, and colors differentiate entity types: light green nodes in (**B**), (**C**), (**E**), (**F**), and (**G**) denote AMTs; brown nodes in (**A**) and (**D**) represent diseases; orange nodes in (**B**), **(D**), (**E**), and (**G**) indicate drugs; purple nodes in (**C**) depict patients; red nodes in (**D**) show drug combinations; pink nodes in (**E**) stand for drug effects; gray nodes denote drug characteristics; dark green nodes in (**F**) represent drug categories; blue nodes in (**G**) and (H) signify drug effects; pink nodes in (**H**) indicate treatment methods; and yellow nodes in (**I**) depict disease stages. The contents that are hidden within entities are explained in Supplementary Appendix 2).

The beta-blocker case encompasses 11 concept nodes, including “AMT, disease, drug, patient, drug combination, drug effect, drug characteristics, MT, treatment method, and disease staging.” As outlined in Section 2.4, the ontology construction model establishes multiple ontology node relationships, including disease-disease and drug-AMT. The ontology knowledge concepts and relationships associated with beta-blockers in EKG-CMG are illustrated in Figure 12.

### 3.4 Mining cardiovascular medication knowledge and reasoning application based on the EKG-CMG

#### 3.4.1 Multi-hop hierarchical knowledge discovery of multiple disease for a single drug

The same drug may exert therapeutic or ameliorative effects across various diseases, and EKG-CMG facilitates the exploration of this “one drug for multiple uses” phenomenon within the cardiovascular domain. Using beta-blockers as an example, EKG-CMG transforms medication knowledge into a retrieval task by identifying connected components within the knowledge graph related to “drug-disease” relationships, thereby generating a knowledge connectivity graph centered on drug nodes. Within the drug-disease connectivity graph, EKG-CMG uncovers potential associations between drugs and various disease nodes through multi-hop paths, identifying multiple therapeutic effects.

**Figure 13:**
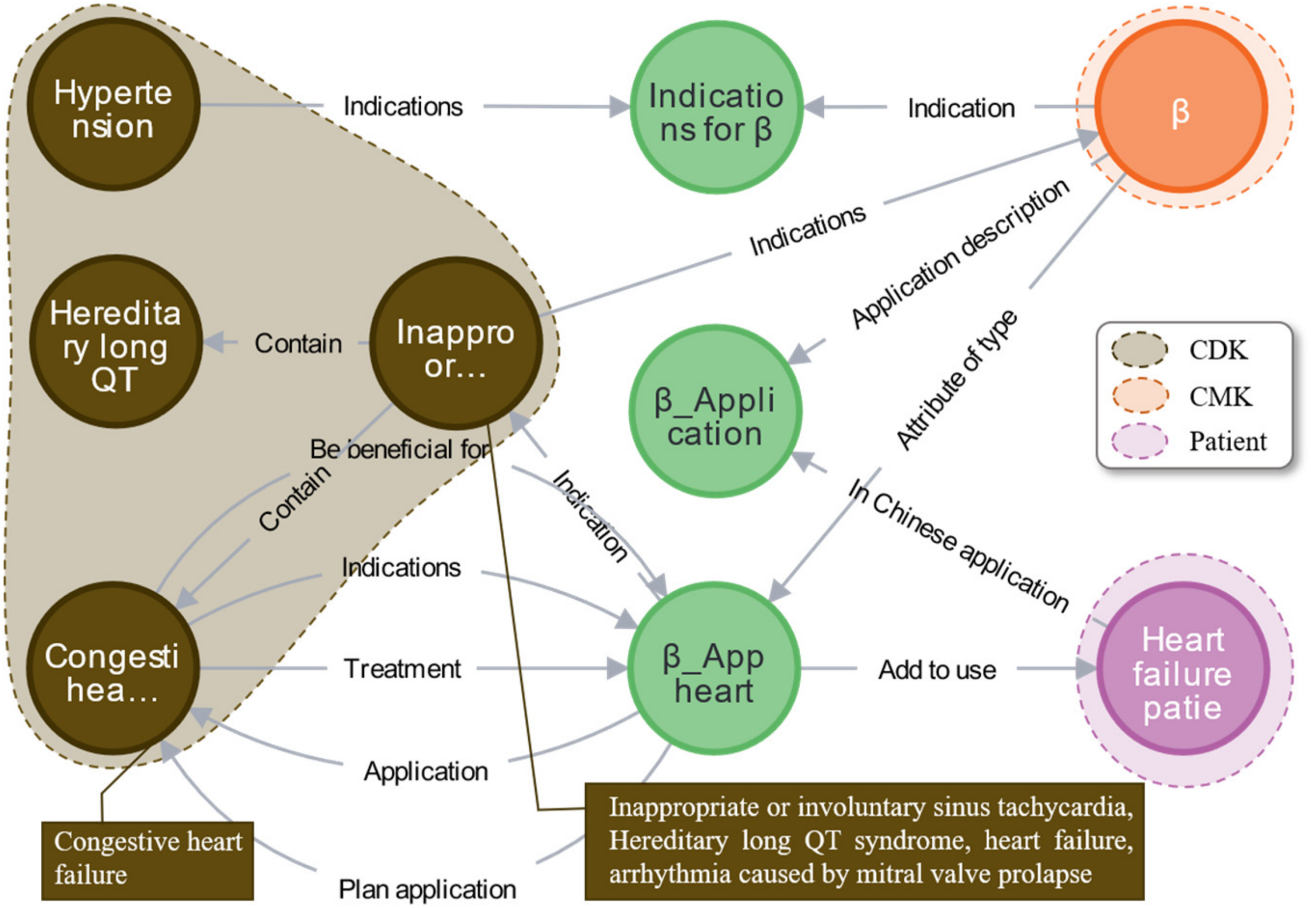
Case analysis of multi-hop hierarchical knowledge discovery for multiple diseases with a single drug.

The specific approach in Supplementary Algorithm 1 (Appendix 3), presents the pseudocode for the “one drug for multiple uses” knowledge reasoning algorithm based on the connectivity graph. Analysis of the connectivity graph for beta-blockers reveals that drugs of the same class exert therapeutic effects across different diseases or symptoms. As illustrated in Figure 13, beta-blockers (indicated by the orange area) not only treat heart failure (pink area) but also show efficacy in managing other cardiovascular diseases (brown area) including hypertension, hereditary long QT, and congestive heart failure. With support from CKG-CMG, all drug indications can be rapidly identified, optimizing clinical diagnosis and treatment decisions.

#### 3.4.2 Mining knowledge of synergistic treatment of multiple cardiovascular drugs

In the clinical medication of cardiovascular diseases, combination medication offers greater synergistic efficacy than single-drug therapy. Using EKG-CMG, the discovery of synergistic treatment knowledge in cardiovascular medication is framed as a connected graph-based intelligent computing problem. With cardiovascular disease as the retrieval starting point, a connected graph generation algorithm traverses relevant medication nodes within EKG-CMG, forming a disease-drug connectivity subgraph for the specified disease.

Because the disease-drug connectivity graph encompasses comprehensive drug information, direct retrieval may introduce numerous redundant nodes, potentially hindering clinicians’ comprehension and decision-making regarding synergistic treatment knowledge. To improve the relevance and accuracy of the retrieval results and reduce the information entropy, we integrate the weights of the medication relationship to optimize the redundant knowledge and use weighted critical path analysis to mine the relationship of drug collaborative treatment. This process references the multi-drug synergistic treatment algorithm for heart failure (Supplementary Appendix 4, Algorithm 2: Pseudocode for the synergistic treatment knowledge mining algorithm based on the weighted critical path).

**Figure 14:**
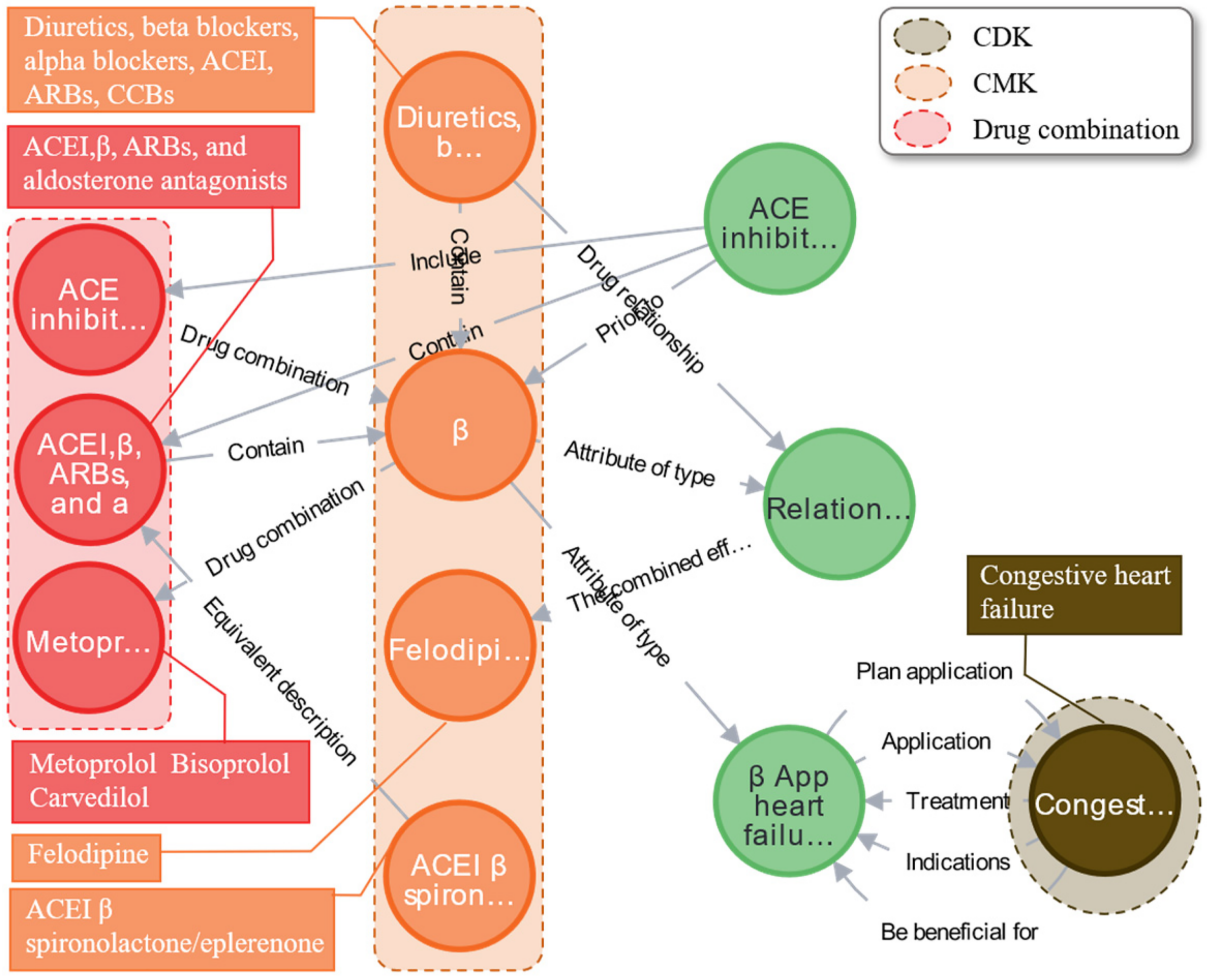
Case analysis of the graph of synergistic therapeutic knowledge of multiple drugs using beta blockers as an example. (The complete content of the relationship called “The combined eff…” is “The combined effect is better than.”).

The study results indicate that the combination of beta-blockers (such as metoprolol, bisoprolol, and carvedilol) with felodipine, represented in the orange area, can produce synergistic therapeutic effects on congestive heart failure, as shown in the brown area. This combination effectively reduces systemic vascular resistance, increases ejection fraction, lowers blood pressure, regulates heart rate, and significantly improves cardiac function, as illustrated in Figure 14. Similarly, these can be combined with drugs such as digoxin or sodium nitroprusside to alleviate rapid heartbeats. Expert evaluations suggest that the synergistic medication knowledge in EKG-CMG can effectively guide clinical combination therapy and support clinicians in developing cardiovascular combination medication plans.

#### 3.4.3 The Mk-MST mining knowledge reasoning for fine-grained precision medication

Precision medicine seeks to enhance healthcare quality through personalized approaches (29). It encompasses targeted prevention, diagnosis, medication, treatment, and monitoring, enabling the formulation of optimal medication plans tailored to each patient. Cardiovascular diseases encompass diverse types, treatment methods, and medication information, presenting challenges due to their complexity. The medication knowledge system for cardiovascular diseases is highly complex, with distinctive characteristics that make it challenging to analyze. In response, we propose a precise medication knowledge-mining approach based on EKG-CMG. This method employs a medication knowledge connectivity graph to generate a Medication Knowledge Minimum Spanning Tree (MK-MST). The MK-MST extracts core and precise medication knowledge—such as medication cycle, frequency, administration methods, precautions, adverse reactions, and dosage—to support clinical decision-making and facilitate precise medication.

Taking Beta-blockers as an example, the MK-MST can analyze precise medication knowledge reasoning (Supplementary Appendix 5 is Algorithm 3: Pseudocode for fine-grained medication knowledge mining using MK-MST). In MK-MST reasoning, beta-blockers can denote a category of cardiovascular medications or specific drugs, including propranolol, metoprolol, carvedilol, bisoprolol, and labetalol. Results indicate that the MK-MST captures core medication information related to beta-blockers. For instance, propranolol is administered in tablet form to treat myocardial infarction and hypertension. As shown in Supplementary Appendix 6 in Figure S1, the green region represents hypertension medication knowledge, indicating an initial dose of 10 mg that may be gradually increased, with a maximum dosage of 200 mg administered 3–4 times daily. The orange region represents medication knowledge for myocardial infarction, with a dosage range of 30–240 mg administered 2–3 times daily.

Additionally, when propranolol treats myocardial infarction, the EKG-CMG can identify medication precautions and potential adverse reactions. For instance, side effects such as sinus bradycardia, atrioventricular block, and hypotension may arise following propranolol administration. Consequently, mining precise medication knowledge from EKG-CMG offers valuable auxiliary support for physicians. Experimental results indicated that expert evaluations found the MK-MST-based precise medication knowledge mining method highly effective in supporting physicians in developing personalized treatment plans. The MK-MST function demonstrates significant clinical potential for enhancing medication management, treatment decision-making, and prescription formulation in cardiovascular care.

#### 3.4.4 Discovery of cardiovascular disease symptom crossover and medication similarity knowledge based on the EKG-CMG

Cardiovascular patients often present with multiple diseases and symptoms, known as “comorbidity” in clinical medicine. Comorbidity is characterized by increased complexity in medication and heightened challenges in treatment decision-making. To address this, we propose a method based on EKG-CMG to discover knowledge about symptom intersection and similarity. First, a knowledge connectivity map encompassing diseases, symptoms, drugs, and treatment methods—the Connectivity Map of Symptom Intersection (CMIS) is generated. CMIS incorporates knowledge of medication similarities for intersecting symptoms, serving as a crucial reference and guide for comorbidity treatment. The proposed method enables physicians to rapidly identify medication knowledge for intersecting symptoms, streamline medication planning, and reduce the complexity of treatment decision-making. For example, with atrial fibrillation and sinus bradycardia, the specific query statements are as follows:

MATCH *p* = (n1)-[*]-(n2)

WHERE (n1: *diseases* OR n1: *symptoms* OR n1: *drugs* OR n1: *treatment methods* AND n1.name IN [‘atrial fibrillation’, ’sinus bradycardia’])

AND (n2: *diseases* OR n2: *symptoms* OR n2: *drugs* OR n2: *treatment methods* AND n2.name IN [‘atrial fibrillation’, ’sinus bradycardia’])

RETURN *p*

Knowledge reasoning results indicate that multiple diseases and symptoms overlap similarly treatment methods may apply to different symptoms. For instance, the brown area in the cardiovascular disease knowledge (CDK) map represents sinus bradycardia and atrial fibrillation, while the blue area signifies the intersection of these two symptoms. Sinus bradycardia is characterized by symptoms such as bradycardia and cardiogenic syncope, while atrial fibrillation presents with bradycardia and frequent premature contractions. Bradycardia is a shared symptom (comorbidity) between the two conditions, with a similarity in the EKG-CMG reasoning indicating β-receptor blockers as unified treatment knowledge, as illustrated in Supplementary Appendix 6 in Figure S2.

In response to the similar phenomenon of comorbidity during disease progression, clinical practice has proposed a comorbidity management approach. Comorbidity management emphasizes a unified baseline treatment for common symptoms across multiple diseases, with medications added or adjusted based on the individual patient’s condition (30). Therefore, clinicians can refer to the symptom intersection and similarity reasoning knowledge within EKG-CMG, integrating it with clinical experience, professional expertise, and the patient’s specific condition to provide scientifically grounded medication guidance.

#### 3.4.5 Comorbidities affect the choice of cardiovascular medication decisions

The complex and interconnected nature of cardiovascular complications makes it challenging to develop effective treatment plans. Treatment plans for complications must consider the patient’s symptoms and drug tolerance. For instance, the beta-blockers are contraindicated for patients with both hypertension and severe tachycardia. For patients intolerant to ACE inhibitors (ACEIs), alternative drugs like angiotensin II receptor blockers (ARBs) can be administered (31). Efficient management and retrieval of comorbidity medication knowledge reduce the decision-making burden on doctors. Consequently, we propose a comorbidity medication treatment reasoning based on EKG-CMG. This approach generates a connectivity graph of comorbidity medication knowledge, the Connectivity Map of Comorbidities (CMC).

During the CMC generation, the same medication node can be traversed multiple times, creating invalid or redundant loops and increasing the retrieval cost of medication knowledge. The medication reasoning outcomes often require multiple rounds of filtering and screening by physicians before clinical application. However, by integrating CMC with shortest path reasoning, the essential for filtering and screening by clinicians is minimized, revealing implicit relationships between comorbidities and medication treatments. Using hypertension and its comorbidities as an example, the medication query statement below:

MATCH (n)

WHERE n.name IN [“*combination*”, “*hypertension*”] OR n.label IN [“*disease*”, “*treatment methods*”, “*drugs*”]

WITH collect(n) AS nodes

UNWIND nodes AS n1

UNWIND nodes AS n2

MATCH path = shortestPath((n1)-[*]-(n2))

WHERE id(n1) <> id(n2)

RETURN DISTINCT path

The CMC results indicate that intravenous beta-blockers can effectively treat isolated hypertension. However, beta-blockers are contraindicated for patients with both hypertension and diabetes, as illustrated in Supplementary Appendix 6 in Figure S3. Experts concur that this feature of EKG-CMG enhances the accuracy, scientific rigor, safety, and effectiveness of clinical treatment decisions. In the clinical diagnosis and management of cardiovascular diseases, comorbidity knowledge mining and reasoning are crucial for optimizing treatment plans. CMC offers significant clinical value by enhancing drug utilization strategies in cardiovascular disease treatment.

#### 3.4.6 Different application of medication knowledge for the same symptom at different stages

Cardiovascular diseases present distinct clinical manifestations and etiologies across various stages. Disease classification, staging, and typology facilitate targeted medication plans to safeguard patient life, health, and organ function. Medication knowledge requirements vary significantly across stages of the same disease, necessitating precise and personalized treatment approaches. Accordingly, based on EKG-CMG, this study infers medication treatment plans for the same symptoms across different stages. First, the Connectivity Map of Disease Stages (CMDS) is generated using the EKG-CMG. Next, knowledge retrieval from CMDS is combined with clinical expert insights to generate stage-specific medication knowledge for the same symptoms. To prevent unnecessary repetitions in loops, we use topological sorting of graph structures to improve semantic retrieval for staged medication treatment plans. For example, the query statement to retrieve graded medication knowledge for hypertension is as follows:

MATCH (n)

WHERE n.name IN [“*Hypertension*”, “*Isolated systolic hypertension*”, “*Simple hypertension*”, “*Hypertensive crisis, hypertensive emergency*”]

WITH collect(n) AS nodes

UNWIND nodes AS n1

UNWIND nodes AS n2

MATCH path = shortestPath((n1)-[*]-(n2))

WHERE id(n1) <> id(n2)

WITH path, [n1, n2] AS endpoints

MATCH (endpoint)-[r]-(otherNode)

WHERE endpoint IN endpoints AND (otherNode: *treatment methods* OR otherNode: *drugs* OR otherNode: *results*)

WITH path, collect (DISTINCT otherNode) AS connectedNodes

RETURN DISTINCT path, connectedNodes

Experimental results indicate that the clinical stage significantly influences treatment options and medication selection. For instance, treatment options and medication choices vary across different hypertension stages. Patients with grade II hypertension are advised to promptly initiate ARNI or diuretic monotherapy. Grade I hypertension typically requires lifestyle interventions; however, if blood pressure remains elevated after 4 to 12 weeks of monitoring, ARNI or diuretic monotherapy should be initiated. In contrast, grade III hypertension requires a markedly different medication approach compared to lower-risk grades I and II, as the same treatments cannot be applied due to the heightened risk of target organ damage. Supplementary Appendix 6 in Figure S4 presents sample search results from EKG-CMG, illustrating that different stages of the same symptom significantly impact fundamental treatment methods and medication selection. EKG-CMG assists clinicians in focusing on classification, staging, type management, and targeted medication strategies for cardiovascular diseases. ARNI is sourced from supplementary guideline data (17), further demonstrating the scalability of the EKG-CMG.

## 4 Discussion

### 4.1 Necessity, breakthrough and advancement of the EKG-CMG research

Cardiovascular diseases pose a significant threat to life and health, showing a trend toward younger, with morbidity, disability, and mortality rates rising annually. Clinical decision-making is crucial in treating cardiovascular, with treatment plans impacting therapeutic outcomes and prognosis management. However, the complexity of cardiovascular and personalized medication makes clinical planning dynamic and challenging.

Existing cardiovascular medication knowledge (CMK) is scattered across medical guidelines and clinical electronic records. They present large volumes of data with discrete, redundant, and unstructured content, hindering doctors’ ability to efficiently understand and apply CMK and increasing the cost of knowledge acquisition. EKG-CMG employs the Neo4j graph database for structured data management, enhancing the availability, efficiency, and convenience management of clinical medication knowledge. Trends in intelligent diagnosis, rapid triage, and high-quality clinical decision support (32, 33) highlight EKG-CMG as a vital tool for advancing these capabilities.

Recently, knowledge graphs have been closely integrated with machine learning, deep learning, and other technologies, making notable progress in medical applications, particularly in drug-gene relationship discovery (34), intelligent diagnostic assistance (35), personalized recommendations (36), decision support (9), and disease prediction (37). These advancements reduce the workload for clinicians and lay a theoretical foundation for advanced medical knowledge mining and reasoning. However, these studies often encounter issues such as incomplete knowledge graphs and low reliability in reasoning results (38). Existing studies primarily utilize explicit knowledge sources (e.g., test data, and medical literature), with limited attention to the implicit experiential knowledge of clinical experts, thereby restricting the effectiveness of knowledge graphs in clinical guidance.

Additionally, most research focuses on mining electronic medical records and general medical information, primarily employing methods like named entity recognition (13), relationship classification (14), and entity disambiguation (15) for knowledge extraction and graph construction, resulting in limited expansion of knowledge mining applications. For instance, Zhao et al. (18) developed a TCM-based knowledge graph for diabetic nephropathy from TCM diagnostic guidelines and consensus. Solely focusing on knowledge acquisition and graph construction, while neglecting longitudinal reasoning into knowledge meaning and logical relationships, reflects a lack of in-depth knowledge analysis and understanding.

Given the vast variety of cardiovascular drugs and the complexity of medication, clinicians face challenges in quickly mastering and retaining essential medication information. Patients, meanwhile, are often curious about medication details yet confused by the complex professional language, making it difficult for them to understand. To address these challenges, the EKG-CMG conducts in-depth research into CMK. Beginning with unstructured and semi-structured data sources, including CMG, expert consensus, and clinical practice experience, the EKG-CMG identifies entities, relationships, and other information to establish standardized and precise medication knowledge.

By constructing EKG-CMG, implicit CMK is comprehensively explored, enhancing a precise understanding of cardiovascular medication at both coarse-grained and fine-grained levels. EKG-CMG not only aids doctors and medical students in mastering medication knowledge more efficiently but also enhances their diagnostic and treatment skills, fostering precise judgment in clinical decision-making. Furthermore, this study provides a theoretical foundation and methodological reference for CMK discovery, supporting the advancement of precision medicine in cardiovascular care.

### 4.2 Importance, professionalism and scalability of precision medication knowledge with the EKG-CMG

#### 4.2.1 Enhancing knowledge on “one drug for multiple uses,” “combination therapy,” and “precision medication.”

Malick et al. (39) utilized a multi-layer knowledge graph to visualize and query gene and protein interactions related to diabetes. Extending this approach, we developed EKG-CMG to explore the phenomenon of “one drug for multiple uses” in cardiovascular. Using a “drug-disease” connectivity graph, we proposed a multi-hop hierarchical knowledge discovery method to retrieve and manage the multiple indications of a single drug. This approach enables rapid identification of the full range of drug indications, facilitating the efficient management and clinical application of “one drug for multiple uses” knowledge. In the future, additional medical data (e.g., genomic data, protein interactions, pathological images) will be integrated into the “drug-disease” connectivity graph of EKG-CMG to further enrich the knowledge and enhance the biological significance of multi-hop hierarchical knowledge discovery.

Combination therapy is often more effective than single-drug therapy, though it results in more complex treatment regimens. This paper proposes a “disease-drug connectivity graph” analysis method based on weighted critical paths to explore knowledge of synergistic therapies. The accuracy of knowledge inference for combination therapy is highly dependent on the quality of relationship weight data; however, to address this limitation, we implemented a dual-expert intervention quality control process and enhanced the study’s practical relevance.

Precision medication knowledge extraction is central to precision medicine. This study introduces MK-MST, a knowledge extraction method based on minimum spanning tree algorithms, to capture precise core medication information, such as dosage cycles, frequency, precautions, and dosage levels. Notably, differences in drug dosage forms can lead to substantial dosage variations; for example, nifedipine controlled-release tablets and drip pills differ significantly in dosage specifications. In the future, we plan to integrate dosage form-specific instruction data into EKG-CMG to enhance its precision and granularity.

#### 4.2.2 Enhancing complex medication knowledge for cross-symptoms, comorbidities, and challenging diseases

Cross-symptom complexity adds significant challenges to medication decision-making. Discovering and mining this knowledge is crucial for supporting the treatment of comorbidities. The CMIS proposed includes information on diseases, symptoms, medication knowledge, and treatment methods, enabling doctors to identify cross-medication and optimize treatment design. We introduce a clinical expert feedback mechanism that integrates doctors’ application feedback into the model to enhance the reliability of cross-symptom medication reasoning, thereby achieving a data enhancement method of “expert knowledge” in the loop. Additionally, the system incorporates a quintuple data access interface for medication knowledge, supporting the integration of individualized patient information such as symptom combinations, genetic profiles, and metabolic characteristics.

Treating comorbidities requires a comprehensive assessment of patient drug tolerance. This paper proposes a reasoning method that integrates CMC and the shortest path to assist doctors in formulating medication treatment plans for comorbidities and reducing the treatment burden of patients with comorbidities. However, research on typical medication cases for complex disease combinations remains limited. Therefore, this study incorporates guidelines, expert consensus, and classic cases through transfer learning to expand EKG-CMG sub-graphs, enriching comorbid medication knowledge and optimizing treatment plans for comorbidities.

Currently, disease staging research primarily focuses on cancer (40–42), and systematic knowledge of staging-based medication for cardiovascular diseases remains limited. The EKG-CMG guideline data includes knowledge of grading, staging, and typology management of hypertension. Using hypertension as an example, we propose a staging medication reasoning method that integrates CMDS and topological sorting to enhance semantic retrieval capabilities for graded medication. In the future, plans include adding “time series nodes” to EKG-CMG to track changes in drug or therapy efficacy at each stage. We will predict treatment outcomes and potential symptom changes using time series data, thus enabling more personalized support for staged medication treatment.

#### 4.2.3 Expanding medication knowledge to additional disease areas

This study allows for an expansion of medication knowledge beyond just the cardiovascular field. Supporting the future inclusion of guidelines for diseases such as hyperglycemia, chronic kidney disease, and sleep apnea. This broader scope will enrich data sources and facilitate the expansion, management, and visualization of medication knowledge across multimodal disease areas.

## 5 Conclusion

This study utilized explicit and implicit knowledge sources, including cardiovascular medication guidelines, expert consensus, and clinical experience, to construct EKG-CMG. Associations between concept ontologies were analyzed and structured within the knowledge base, with relationship hierarchy weights designed to support medication knowledge reasoning. The construction of EKG-CMG facilitated standardized, systematic, and structured, while also enabling visualization of CMK. The EKG-CMG enabled multi-hop hierarchical knowledge discovery via a knowledge graph reasoning algorithm while facilitating the mining of implicit knowledge and revealing internal relationships within clinical CMK. The EKG-CMG construction enhances physicians’ mastery of the CMK, improves clinical diagnosis and prescribing skills, and fosters perceptiveness in medication regimen planning. The EKG-CMG offers clinical guidance for formulating and optimizing medication regimens and provides foundational knowledge and theoretical support for developing intelligent decision-making systems in cardiovascular treatment planning.

## Data Availability

The original contributions presented in the study are included in the article/Supplementary Material, further inquiries can be directed to the corresponding author/s.
